# Investigator initiated trials versus industry sponsored trials - translation of randomized controlled trials into clinical practice (IMPACT)

**DOI:** 10.1186/s12874-021-01359-x

**Published:** 2021-08-31

**Authors:** Anette Blümle, Katharina Wollmann, Karin Bischoff, Philipp Kapp, Szimonetta Lohner, Edris Nury, Kai Nitschke, Jasmin Zähringer, Gerta Rücker, Martin Schumacher

**Affiliations:** 1 Institute for Evidence in Medicine (for Cochrane Germany Foundation), Faculty of Medicine and Medical Center, University of Freiburg, Breisacher Str. 86, 79110 Freiburg, Germany; 2 Clinical Trials Unit, Faculty of Medicine and Medical Center, University of Freiburg, Elsässer Straße 2, 79110 Freiburg, Germany; 3 Cochrane Hungary, Clinical Centre of the University of Pécs, Medical School, University of Pécs, Pécs, Hungary; 4 Institute of Medical Biometry and Statistics, Faculty of Medicine and Medical Center, University of Freiburg, Stefan-Meier-Str. 26, 79104 Freiburg, Germany

**Keywords:** Randomized controlled trials as topic, Registries, Access to information, Evidence-based medicine, Publishing, Systematic reviews as topic, Practice guidelines as topic, Knowledge translation, Health impact assessment, Clinical decision-making

## Abstract

**Background:**

Healthcare decisions are ideally based on clinical trial results, published in study registries, as journal articles or summarized in secondary research articles. In this research project, we investigated the impact of academically and commercially sponsored clinical trials on medical practice by measuring the proportion of trials published and cited by systematic reviews and clinical guidelines.

**Methods:**

We examined 691 multicenter, randomized controlled trials that started in 2005 or later and were completed by the end of 2016. To determine whether sponsorship/funding and place of conduct influence a trial’s impact, we created four sub-cohorts of investigator initiated trials (IITs) and industry sponsored trials (ISTs): 120 IITs and 171 ISTs with German contribution compared to 200 IITs and 200 ISTs without German contribution. We balanced the groups for study phase and place of conduct. German IITs were funded by the German Research Foundation (DFG), the Federal Ministry of Education and Research (BMBF), or by another non-commercial research organization. All other trials were drawn from the German Clinical Trials Register or ClinicalTrials.gov. We investigated, to what extent study characteristics were associated with publication and impact using multivariable logistic regressions.

**Results:**

For 80% of the 691 trials, results were published as result articles in a medical journal and/or study registry, 52% were cited by a systematic review, and 26% reached impact in a clinical guideline. Drug trials and larger trials were associated with a higher probability to be published and to have an impact than non-drug trials and smaller trials. Results of IITs were more often published as a journal article while results of ISTs were more often published in study registries. International ISTs less often gained impact by inclusion in systematic reviews or guidelines than IITs.

**Conclusion:**

An encouraging high proportion of the clinical trials were published, and a considerable proportion gained impact on clinical practice. However, there is still room for improvement. For publishing study results, study registries have become an alternative or complement to journal articles, especially for ISTs. IITs funded by governmental bodies in Germany reached an impact that is comparable to international IITs and ISTs.

**Supplementary Information:**

The online version contains supplementary material available at 10.1186/s12874-021-01359-x.

## Background

Decisions in healthcare are ideally built on three pillars, the experience of the clinician, the wishes and values of the patient, and the best available external evidence, i.e. results from clinical research [1]. Available, findable and accessible clinical research results are mandatory for a successful transfer of this knowledge into evidence-based practice and further research [2]. Beside research results, also information about detailed study methods is important, since only they allow to appraise the validity, reliability and applicability of clinical evidence to clinical practice [3].

It has long been known that only a part of the clinical studies conducted ultimately reach the stage of full publication in peer-reviewed journals [4]. For example, more than half of the study results presented as an abstract at scientific meetings fail to be published as a full-text article [5]. Thus, important study information cannot be considered for health care decisions and further research planning, which in turn could expose patients and future study participants to unnecessary risks [6]. Systematic reviews and meta-analyses can come to an erroneous overall effect estimate and conclusion when unpublished data cannot be considered [7]. If experiences and results obtained from trials are not disseminated, they are not only lost for health care, but also for further research. Moreover, personnel resources and scarce research funds are badly invested or even wasted.

An important step for increasing both, the transparency in research and the visibility of unpublished studies was the implementation of study registries as well as the call for prospective study registration by several research organizations [8–10]. In Germany, funding organizations such as the German Research Foundation (Deutsche Forschungsgemeinschaft, DFG) and the Federal Ministry of Education and Research (Bundesministerium für Bildung und Forschung, BMBF) require the registration of the trial in a public registry and publication of the trial protocol following grant approval [11, 12]. Prospective study registration is a major step forward, but it is equally important to make the results of a trial publicly available, which is possible through study registries. However, even several years after these urgent calls for a prospective study registration, there are still trials that are not included in a study registry [13]. Thus, unpublished studies and their results are difficult to identify.

In recent years, several authorities and research organizations became aware of the problems arising from withholding study results. The World Health Organization (WHO), the World Medical Association (WMA) and the All Trials initiative [14], have alerted that it is unethical to conduct human research without subsequently publishing the results. They also pointed out that vast financial resources spent on clinical research are wasted when research results are not published. Hence, these research organizations took various steps to prevent incomplete, biased or non-reporting of research results [15].

To the best of our knowledge, it is still unclear under what conditions expenses are invested to support clinical trials pay off in a way that the findings have an impact on healthcare decisions. As an order of magnitude, in 2018 the German Research Foundation (DFG) alone spent 22 Million euros for the conduction of 47 trials within their clinical trials program [16]. Trial discontinuation could be identified as one factor for non-publication of clinical trials [17]. Another major step forward would be to identify trial specific risk factors for non-publication or for having no impact on medical practice.

The aim of this project was to examine the transfer process of clinical trial information into medical practice. First, we determined the proportion of the trials that were published, the type (methods and/or results) and place (as journal article, register entry) of published information and the proportion of trials cited by secondary research articles (reviews and/or clinical guidelines). We then analyzed whether there is an association of pre-defined study characteristics (sponsoring/funding, study phase, drug/non-drug intervention, number of participants, number of primary outcome, medical field) with publication or use by secondary research articles.

## Methods

The rationale and design of this project is described in detail in a previous publication [18].

### Study cohort

In brief, we set up a MS Access database consisting of 691 trials (hereafter referred to as study cohort). Eligible for inclusion were clinical trials that were conducted at multiple study sites, were randomized controlled (RCTs), investigated drugs or non-drugs, started in 2005 or later and were completed by the end of 2016. To find out whether sponsorship/funding or place of conduct influence a trial’s impact, we created and compared sub-cohorts of investigator initiated trials (IITs) and industry sponsor trials (ISTs) with and without German contribution (Table 1). For the IIT-sub-cohort “Public Germany” we included trials funded by the DFG and BMBF (Public Germany gov), which we retrieved from the funder’s databases “German Project Information System” (GEPRIS) of the DFG and the website of the BMBF [19, 20]. These IITs served as basis for the determination of the eligibility criteria for the trials to be included in the comparison sub-cohorts. The largest trial of the reference sub-cohort included 4005 participants so that we only considered trials up to this sample size for inclusion in the other sub-cohorts. To achieve a reasonable number of German IITs, we complemented the reference sub-cohort by an equal number of IITs funded by other German non-commercial organizations (Public Germany other), which we randomly drew from the trials registries ClinicalTrials.gov and German Clinical Trials Register (DRKS) (Table 1). Trials included in the sub-cohort Commercial Germany were also drawn from these two registries, whereas trials included in the international sub-cohorts (Public International and Commercial International) were solely drawn from ClinicalTrials.gov.

**Table 1:** Characteristics of included trials

Characteristics	IIT Public Germany gov No. of trials (%)	IIT Public Germany other No. of trials (%)	IIT Public Germany (total) No. of trials (%)	IIT Public International No. of trials (%)	IST Commercial Germany No. of trials (%)	IST Commercial International No. of trials (%)	Total No. of trials (%)
**Total**	60	60	120 (100)	200 (100)	171 (100)	200 (100)	691 (100)
**Registered in**^**a**^							
ClinicalTrials.gov	32 (53)	16 (27)	48 (40)	200 (100)	158 (92)	200 (100)	606 (88)
DRKS^b^	14 (23)	48 (80)	62 (52)	–	19 (11)	–	81 (12)
ISRCTN^c^	27 (45)	5 (8)	32 (27)	3 (1)	–	–	35 (5)
EudraCT^d^	40 (67)	10 (17)	50 (42)	18 (9)	88 (52)	33 (17)	189 (27)
**Study status**							
Completed	43 (72)	59 (98)	102 (85)	200 (100)	170 (100)	200 (100)	672 (97)
Prematurely ended	12 (20)	1 (2)	13 (11)		1 (< 1)		14 (2)
Still ongoing^e^	5 (8)	–	5 (4)				5 (< 1)
**Collaboration**							
International	19 (32)	7 (12)	26 (22)	44 (22)	71 (42)	69 (35)	210 (30)
National	40 (66)	53 (88)	93 (78)	156 (78)	100 (58)	131 (65)	479 (69)
Unclear	1 (2)	–	1 (< 1)	–	–	–	2 (< 1)
**Study size (Median = 150)**							
> 150	46 (76)	28 (47)	74 (62)	81 (40)	74 (43)	115 (58)	344 (50)
≤ 150	13 (22)	32 (53)	45 (38)	119 (60)	97 (57)	85 (42)	346 (50)
Unclear	1 (2)	–	1 (< 1)	–	–	–	1 (< 1)
**Number of primary outcome(s)**							
0	–	–	–	–	1 (1)	–	1 (< 1)
1	44 (73)	44 (73)	88 (73)	152 (76)	122 (71)	133 (67)	495 (72)
> 1 (range 2–36)	16 (27)	16 (27)	32 (27)	48 (24)	48 (28)	67 (33)	195 (28)
**Study phase drug trials**^**f**^							
Total	41 (68)	15 (25)	56 (47)	93 (47)	93 (54)	93 (47)	335 (48)
2	9 (15)	5 (8)	14 (12)	23 (12)	23 (13)	23 (12)	83 (12)
3	20 (33)	7 (12)	27 (22)	45 (23)	45 (26)	45 (23)	162 (23)
4	12 (20)	3 (5)	15 (13)	25 (13)	25 (15)	25 (13)	90 (13)
**Study phase non-drug trials**^**g**^							
Total	19 (32)	45 (75)	64 (53)	107 (53)	78 (46)	107 (53)	356 (52)
A	–	9 (15)	9 (7)	15 (7)	11 (7)	15 (7)	50 (7)
B	16 (27)	33 (55)	49 (41)	82 (41)	43 (25)	82 (41)	256 (37)
C	3 (5)	3 (5)	6 (5)	10 (5)	24 (14)	10 (5)	50 (7)

^a^ Several trials were registered in more than one trials registry, i.e. numbers do not sum up to the total numbers (100%); ^b^DRKS: German Clinical Trials Register; ^c^ISRCTN: International Standard Randomized Controlled Trials Number registry; ^d^EudraCT: European Union Drug Regulating Authorities Clinical Trials Database; ^e^Status as of 24 April 2020; ^f^15 drug trials of phase 2–3 were counted as phase 2; 24 non-drug trials of phase A-B were counted as phase A; ^g^In the sub-cohort “Commercial Germany”, we included all non-drug trials available in the study registries, resulting in slightly differing distributions of study phases among the 4 sub-cohorts

To minimize possibly biasing study characteristics, we aimed to generate comparable sub-cohorts by balancing for effects of the study phase and the location of participating study sites (proportion of German study sites). According to the distribution given in the sub-cohort Public Germany, we balanced the three comparison sub-cohorts Public International, Commercial Germany and Commercial International for the study phase, of both drug trials and non-drug trials, and the sub-cohort Commercial Germany additionally for the proportion of German study sites on all study sites (Table 1).

We independently double-extracted the pre-defined study characteristics such as sample size, study phase, number of pre-defined primary outcomes, and medical fields [21] from the study registries, as we were interested in whether they were associated with research impact. For further details concerning the project methods please refer to the methods paper [18].

### Identification of corresponding publications

For each trial, we identified related publications and classified them according to the published trial information: method article only (solely the study methods are described in detail), result article only (study results are described and usually the methods very briefly), and both. This classification allowed us to determine what kind of study information was used in secondary research articles and clinical guidelines.

#### Search strategy: sources where journal articles were identified

First, we searched for publications in different biomedical databases and other sources using an incremental search strategy (Additional file 2). As search terms, we combined various study information such as the registry identification number, study title, acronym, PICO-aspects, and/or name of applicant or principal investigator. Searches for primary study reports were conducted between 6 February 2018 and 30 August 2018. We then downloaded the references of all identified published articles into an Endnote database. We considered full articles reporting a trial’s methods and/or results. We also downloaded all the study protocols we came across during our literature search.

#### Trial information in study registries

Beside study registration, publishing trial results in study registries is required since several years [14, 22, 23]. In the DRKS, study related documents can be attached or linked to the trial record. In ClinicalTrials.gov, results can be entered directly into the trial record as a separate register tab or are automatically searched and attached by the study registries themselves [24].

In addition to the publication as a journal article, we determined whether or not study information was available in study registries. Beside their registration in ClinicialTrials.gov and DRKS, 189 (27%) of the trials were additionally registered in EudraCT and 35 (5%) in the ISRCTN registry. Trials with results available in study registries are hereafter referred to as “results in registries” [18].

### Definition

Hereinafter, we use the following definitions: for publications in journal articles we use the expression “published articles”. We distinguish between articles solely concerning a trial’s methods, called “method articles”, and articles also reporting study results (“result articles”). Beside publication as journal article, results can be published in study registries; in this case, we use the expression “results in registries”. For published trial results, i.e. as result article or as results in registries, we use the general term “published results”.

### Identification of secondary research articles citing primary published articles

To assess the research impact of the included trials, we investigated whether or not published articles were cited by secondary research articles, i.e. systematic reviews/meta-analyses and clinical guidelines.

#### Systematic reviews

For each published article, we downloaded all references listed under the functions “Cited by” in PubMed and “Times Cited” in Web of Science. To identify the systematic reviews and meta-analyses among the citing articles, we matched their Digital Object Identifier (DOI) with the record-DOIs included in the database Epistemonikos, which can be considered as the “largest source of systematic reviews relevant for health-decision making” [25]. Epistemonikos includes references of four categories: broad syntheses, systematic reviews, structured summaries and primary studies. In our project, we focused on references classified as systematic reviews or broad syntheses. Both categories are hereinafter referred to as “systematic reviews” (SRs).

If a DOI of a citing article was found in Epistemonikos, the publication type was verified and the citing article labelled as systematic review. We then manually assessed how the published articles were used and where they were cited in the systematic reviews/meta-analyses:

- General information or methods of the published article were used and cited in the systematic review, e.g. in the introduction or discussion section,
- Study results reported in the published article were included in the systematic review/meta-analyses or
- Study results reported in the published article were not included in the systematic review/meta-analyses, e. g. not meeting eligibility criteria.

#### Clinical guidelines

The ultimate step for a successful implementation of trial’s results in medical practice is their inclusion in clinical practice guidelines. To identify these, we manually searched in the clinical guidelines databases TRIP [26], NICE evidence search [27] and AWMF (Association of the Scientific Medical Societies) [28]. We searched for clinical guidelines citing the trial publications. As search terms, we used (parts of) the title and the name of the first author of the published articles as well as the corresponding systematic review/meta-analysis; to identify guidelines citing results published in registries, we searched with the register identification number. The search period for guidelines citing the published articles was between December 2018 and March 2019, for guidelines including systematic reviews between April and August 2019, and for the registry identifier in February 2020. For each identified clinical guideline, we retrieved the full text and verified the citations.

### Data collection

We extracted the following information about the publications into an Access database: 1) whether or not study results were reported in study registries, 2) bibliographic information of included publications and content (method article or result article), 3) bibliographic information of citing systematic reviews/meta-analyses, and 4) bibliographic information of citing guidelines.

### Semi-automatic tool

Within this project, one author (KN) developed a semi-automatic tool (called DoiScout) that facilitates large-scale literature searches and citation analyses in order to carry out extensive literature searches based on internet search engines more time-efficiently.

DoiScout automatically identifies primary published articles that reference a particular study registry ID (e.g. NCT02179424). Bibliographic information about the identified articles is extracted and presented in a list that is formatted in a way that allows passing on the information to other software programs for further processing.

A second feature refers to citation analysis. Search engines behind platforms such as PubMed (www.pubmed.gov) and Web of Science (www.webofknowledge.com) can be used to identify other articles, e.g. primary research articles, systematic reviews and clinical guidelines that cite a given article. DoiScout extracts the bibliographic information of the citing articles and provides it to the user in a workable format. In addition, DoiScout can be used to identify articles citing the citing articles of the original source. This can be done for any pre-specified citation depth, thus providing a comprehensive overview of the extent of a project’s academic impact.

The program of the DOIScout and a manual describing the features in more detail are available via the GitHub platform [29].

### Data analysis

We used queries in MS Access 2010TM and tabulation in Microsoft Excel 2010 to obtain standard descriptive statistics. Multivariable logistic regression was used to determine the association of study characteristics with the probability of a trial to be published, cited by systematic reviews and included in guidelines. Based on the reference sub-cohort Public Germany, it was carried out for the other sub-cohorts, for study phase, number of participants, and number of primary outcomes. For time to publication, multivariable Cox regression was used to account for study characteristics. For distinguishing between first publication in a journal or in a registry, a competing risk model was used and Aalen-Johansen estimates of the cumulative incidence functions are presented [30].

## Results

### Definitions

The term “published trials” is used when “method articles”, “result articles” or “results in registers” are available. Results of a trial can be published as “result article” in a journal or as “results in registries”, while methods are always published as “journal article”. We first described the proportion of publication types for the total cohort and then for the different sub-cohorts. If not mentioned otherwise, all percentages of trials given for the entire cohort are calculated on the basis of the included 691 trials. Percentages given for the sub-cohorts are based on the number of trials in each sub-cohort. Minor differences in summed percentages derive from rounding to full integer.

#### Proportion of published trials

**Fig. 1:**
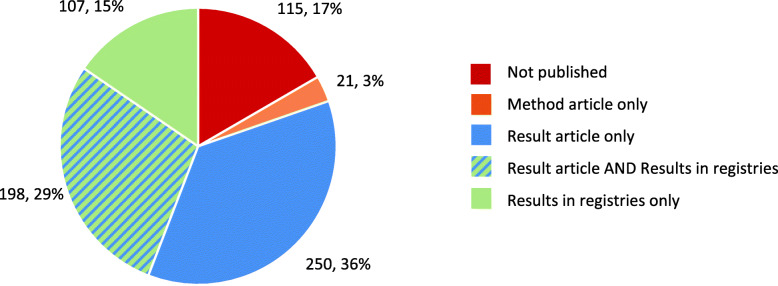
Proportion of published trials and type of publication for the whole cohort (*n* = 691). Please refer also to Table 2

For our whole cohort, 576 (83%) of the 691 trials included were published as a method article or a result article in a medical journal and/or the trial results were made available in study registries; results were available for 555 (80%) of the trials (Fig. 1). For 107 (19%) trials, results were solely published in a registry.

#### Trials published as journal article

##### Cohort

For 472 (68%) of our 691 trials, we identified 947 corresponding published journal articles (Table 2, Additional file 3). Out of 448 (65%) trials, 843 result articles were published. For 100 (15%) trials, 104 method articles, without presenting any trial results, were found. For 372 (54%) trials, only a result article was available. We found both, a method article as well as a result article for 76 (11%) of the trials. For three trials with a method article, results were published only in registries. No results were published for 21 (3%) trials with a method article, neither in a journal article nor in a registry.

**Table 2:** Proportion of published trials per sub-cohort and type of publication (total: *n* = 691)

	IIT Public Germany gov No. of trials (%)	IIT Public Germany other No. of trials (%)	IIT Public Germany (total) No. of trials (%)	IIT Public International No. of trials (%)	IST Commercial Germany No. of trials (%)	IST Commercial International No. of trials (%)	Total No. of trials (%)
**Total trials**	60	60	120 (100)	200 (100)	171 (100)	200 (100)	691 (100)
**Proportion of published trials**							
Published	48 (80)	44 (73)	92 (77)	174 (87)	147 (86)	163 (82)	576 (83)
95% CI	68–88	60–84	68–84	82–91	80–91	75–87	80–86
Not published	12 (20)	16 (27)	28 (23)	26 (13)	24 (14)	37 (19)	115 (17)
**Type of publication; trials published as**							
Journal article	48 (80)	42 (70)	90 (75)	169 (85)	113 (66)	100 (50)	472 (68)
95% CI	68–90	57–81	66–83	79–89	59–73	43–57	65–72
Method article	31 (52)	15 (25)	46 (38)	41 (21)	10 (6)	3 (2)	100 (15)
95% CI	38–65	15–38	30–48	15–27	3–11	0–4	12–17
Result article	34 (57)	38 (63)	72 (60)	163 (82)	113 (66)	100 (50)	448 (65)
95% CI	43–69	50–75	51–69	75–87	59–73	43–57	61–68
Results in registries	3 (5)	2 (3)	5 (4)	65 (33)	101 (59)	134 (67)	305 (44)
95% CI	1–14	0–12	1–10	26–40	51–67	60–74	40–48
Published results	35 (58)	40 (67)	75 (63)	170 (85)	147 (86)	163 (82)	555 (80)
**Combinations**							
Result as article AND in registries	2 (3)	0	2 (2)	58 (29)	67 (39)	71 (36)	198 (29)
Method AND Result article	17 (28)	11 (18)	28 (23)	35 (18)	10 (6)	3 (2)	76 (11)
Method article, no published results	13 (22)	4 (7)	17 (14)	4 (2)	0	0	21 (3)
**Trial information in registries**							
Publ. ref. total	35 (58)	35 (58)	70 (58)	104 (52)	56 (33)	63 (32)	293 (42)
95% CI	50–71	45–71	49–67	45–59	26–40	25–38	39–46
Publ. ref. of result article	27 (45)	30 (50)	57 (48)	102 (51)	55 (32)	62 (31)	276 (40)
95% CI	32–58	37–63	38–57	44–58	25–40	25–38	36–44

For 98% (438 of 448) of the trials with published results, the pre-defined primary outcome was reported in the result article.

The publication frequency, i.e. the number of published articles per trial, is shown in Additional file 4. Many trials (284, 60%) were published solely in one journal article. In the remaining trials, multiple publication was highly represented. For example, only 8% of the trials generated 29% of the publications, resulting in an average publication frequency of 7.0 (median 6) publications per trial.

##### Sub-cohorts

**Fig. 2:**
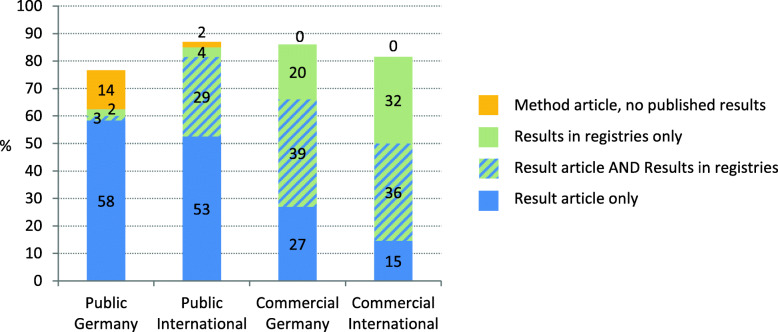
Proportion of published trials and type of publication per sub-cohort. Please also refer to Table 2

For the sub-cohorts, the proportion of trials published varied between 77 and 87% (Fig. 2). Compared to the sub-cohort Public Germany (77%), the probability of a trial to be published is higher for the sub-cohorts Public International (87%), Commercial Germany (86%) and Commercial International (82%) (Table 2). The publication of results ranged between 63% for Public Germany (58% for Public Germany gov and 67% Public Germany other) and 86% for Commercial Germany.

Obvious differences exist between the sub-cohorts regarding the type of publication. IITs were more often published as a journal article than ISTs (Table 2). Especially method articles were more present for IITs (Public Germany 38%, Public International 21%) than for the IST-sub-cohorts (Commercial Germany: 6%; Commercial International: 2%). Compared to the German sub-cohorts, results were more often published as a journal article for Public International trials and less often for Commercial International trials. Looking at the number of publications per trials, multiple publications were more common in IITs (Germany: 33%, International: 32%) compared to ISTs (Germany: 17%, International 14%).

#### Trial information available in study registries

##### Cohort

For 293 (42%) of the 691 included trials, at least one reference to a corresponding journal article was reported in the study registry and/or a link to the original publication source or a database was provided. This means that 62% (293 of 472) of all published journal articles could be found in study registries (Table 2).

Information on results was available for 449 (65%) trials. For 305 (44%) trials, results were directly included in a study registry and for 276 (40%), a reference to a result article was reported. For 132 (19%) trials, both sources were present and for 144 (21%) solely a reference of a result article.

##### Sub-cohorts

The proportion of trials with a reference or link to the journal article was with 58 and 52% higher in the Public sub-cohorts than in the Commercial sub-cohorts with 33 and 32% (Table 2). Results in registries ranged between 4 and 67%. The proportion of IST with results in registries was higher than for IITs.

For Public Germany, only 4% of the trials had results in registries. This small percentage can be explained by the fact that most of those trials derived from the DRKS register (summarized data, see Table 2 for more details), where results cannot directly be entered.

For the three other sub-cohorts, between 29 and 39% of the trials have results published in both registries and as journal articles and for 20 and 32% of the commercial sub-cohorts, results were solely available in registries.

#### Study characteristics associated with publication of results

The multivariable analysis confirmed our findings regarding publication probability for the sub-cohorts. It also showed that additional study characteristics are associated with the probability to be published: drug trials were published more often than non-drug trials, larger trials more often than smaller trials and trials with more than one primary outcome more often than trials with one primary outcome (Additional file 6).

Each trial was allocated to one of 23 pre-defined medical fields (Additional file 6). In our cohort, the median number of trials per medical field was 25 and ranged between 2 (anaesthesiology) and 104 (surgery), the proportion of trials published ranged between 87 and 25%. Statistically significant differences were only found for medical fields with a sufficient number of trials (≥ 39): higher publication rates were found for neurology (87%) and psychiatry/psychotherapy (84%), lower for surgery (64%) and ophthalmology (25%). Due to the limited number of trials per medical field, an analysis for significant differences was not appropriate for the sub-cohorts. Further details on publication and impact are presented in the chapter “Overall impact”.

### Time to publication

The median time to any publication as a journal article or in a study registry, including method papers, was 4.07 years (95% CI: 3.79–4.33). If only counting result papers, the median time was longer (4.67 years, 95% CI: 4.36–5.03). The median time for any type of article (including method papers) to be published in a journal was 5.19 years (95% CI: 4.83–5.82); if only result articles were counted, the median was 6.09 years (95% CI: 5.66–6.62).

**Fig. 3:**
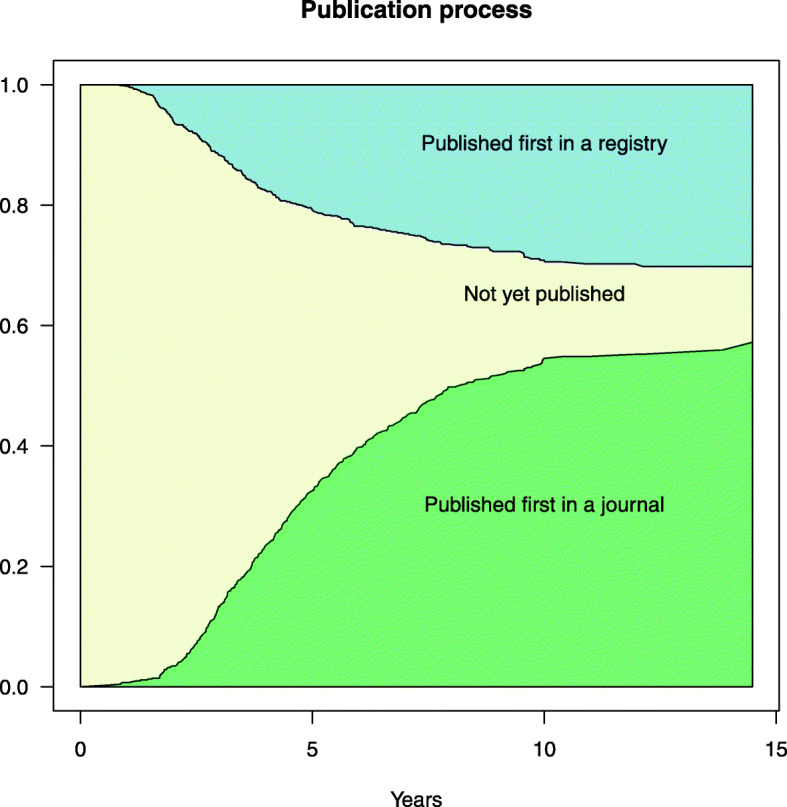
Cumulative incidence functions (Aalen-Johansen estimates)

We analyzed the time to first publication of study results in a journal or in a registry also in the framework of a competing risk model. This was visualized as Aalen-Johansen estimators in a stacked probability plot (Fig. 3). The result shows that for the majority of studies (about 52%) the first publication was found in a journal, while about 28% of studies were first published in a study registry.

**Fig. 4:**
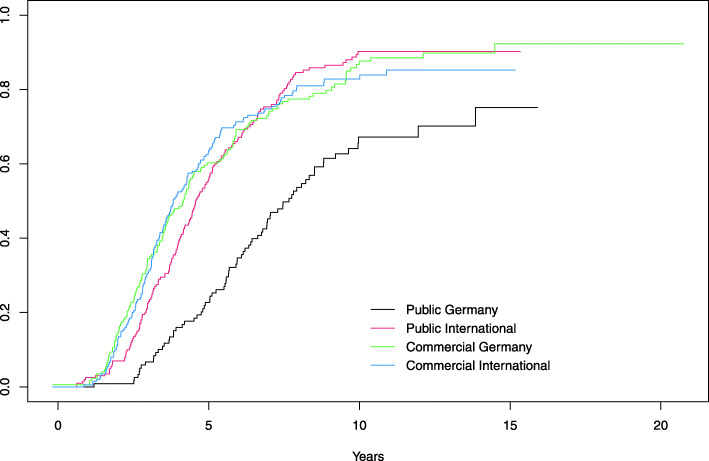
Kaplan-Meier estimates of the cumulative distribution function for time to publication of results, grouped by sub-cohort

Compared to Public Germany trials, results were published earlier for trials of the other sub-cohorts (Table 3, Fig. 4). Furthermore, drug trials were published earlier than non-drug trials and larger trials earlier than smaller. In our cohort, we did not find an association of time to publication with the number of primary outcomes (1 or more than 1).

**Table 3:** Time from study start to publication of results, either in a registry or journal

Covariates	Hazard ratio	95% CI	***p***-value
Intercept	1		
IIT Public International	2.243	1.703–2.956	*P* < 0.001
IST Commercial Germany	2.343	1.770–3.112	*P* < 0.001
IST Commercial International	2.332	1.761–3.072	*P* < 0.001
Non-drug trials versus drug trials	0.838	0.707–0.992	*P* < 0.05
Study size: *n* > 150 versus n ≤ 150	1.215	1.023–1.442	*P* < 0.05
Number of primary outcome(s): > 1 versus 1	1.141	0.939–1.387	n.s.

Multivariable analysis with estimated covariate effects for sub-cohorts, type of intervention, study size, number of primary outcomes. Hazard ratio with 95% confidence intervals. The intercept stands for the combination of IIT Public Germany, drug trial, *n* ≤ 150 and one primary outcome.

### Impact: proportion of trials included in systematic reviews and guidelines

#### Systematic reviews

One measure of impact of a trial is the citation of their journal articles by systematic reviews. In 2631 systematic reviews, 599 of 947 (63%) published journal articles deriving from our trials were cited. Overall, we identified 3429 citations in the reviews, as reviews cited more than one of our journal articles (Additional file 7).

##### Cohort

The 599 articles cited by systematic reviews corresponded to trials (Table 4). Out of those, 27% were cited by only one systematic review; 73% by more than one. It is notable that 15% of the published articles were cited by 10 or more systematic reviews (Additional file 8). The median number of citing systematic review(s) per trial was 4 (range 1 to 99; mean = 4.1).

**Table 4:** Proportion of trials (*n* = 691) cited by systematic reviews per sub-cohort and type of publication

Trials cited by SR	IIT Public Germany gov No. of trials (%)	IIT Public Germany other No. of trials (%)	IIT Public Germany (total) No. of trials (%)	IIT Public International No. of trials (%)	IST Commercial Germany No. of trials (%)	IST Commercial International No. of trials (%)	Total No. of trials (%)
**Total trials**	60	60	120 (100)	200 (100)	171 (100)	200 (100)	691 (100)
Trials in SR	41 (68)	30 (50)	71 (59)	125 (63)	89 (52)	75 (38)	360 (52)
95% CI	55–80	37–63	50–68	55–69	44–60	31–45	48–56
Trials with method article in SR	25 (42)	7 (12)	32 (27)	31 (16)	5 (3)	2 (1)	70 (10)
Trials with method article only in SR	12 (20)	4 (7)	16 (13)	8 (4)	1 (1)	0	25 (4)
Trials with result article in SR	29 (48)	26 (43)	55 (46)	117 (59)	88 (51)	75 (38)	335 (48)
95% CI	35–62	31–57	37–55	51–65	44–59	31–45	45–52
Trials with method AND result article in SR	13 (22)	3 (5)	16 (13)	23 (12)	4 (2)	2 (1)	45 (7)
**Use in SR**							
Trials included in SR	31 (52)	21 (35)	52 (43)	107 (54)	84 (49)	66 (33)	309 (45)
95% CI	38–65	23–48	34–53	46–61	41–57	27–40	41–48

Similar proportions were found for the subgroup of result articles (529 of 843; 63%) and the corresponding trials (335; 48%). Of the 104 method articles, 70 (67%) method articles corresponding to 70 (10%) trials were cited by a systematic review.

We not only examined whether retrieved publications were cited in systematic reviews but also how they were used (excluded, included or used otherwise). As publications included in secondary research articles are more likely to influence clinical practice than excluded publications, this analysis is important for the assessment of the impact of trials. Of the citations in systematic reviews, 69% (2374 from 3429) were included and correspond to 45% (309 of 691) trials (Table 4), 6% (190 of 3429) were excluded and 25% (865 of 3429) were used otherwise. Nevertheless, 69 of the 87 trials with excluded publications in reviews had included publications in other reviews. For the remaining 18 trials, only exclusions were found. Frequently stated reasons for the exclusion of publications were that cohorts failed to meet the eligibility criteria and did not report the data of interest.

##### Sub-cohorts

**Fig. 5:**
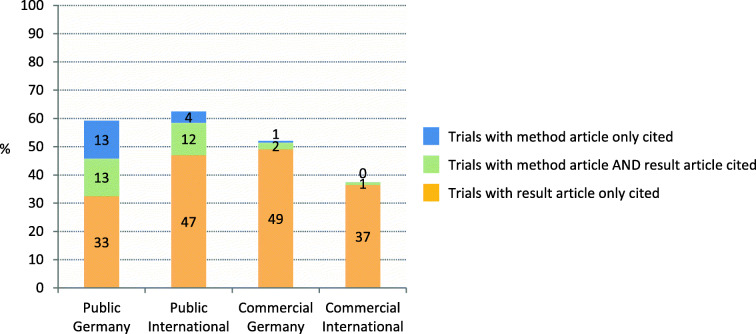
Proportion of trials cited by systematic reviews

For the public sub-cohorts and for Commercial Germany, citation by systematic reviews ranged between 52 and 63% and was higher than in Commercial International with 38% (Fig. 5 and Table 4). This difference might be explained by the lower proportion of trials published as journal articles in ISTs (compare Fig. 2). Furthermore, a relevant proportion of articles cited by systematic reviews were method articles, which were rare in ISTs but mainly present in IITs.

#### Study characteristics associated with citation by systematic review

The multivariable analysis confirmed the significantly lower representation of Commercial International trials in systematic reviews compared to the other sub-cohorts. Both, the type of intervention and the number of primary outcomes are not associated with the inclusion probability, whereas larger trials are significantly more often included in reviews than smaller trials (Additional file 9).

#### Trials included in clinical guidelines

##### Cohort

**Fig. 6:**
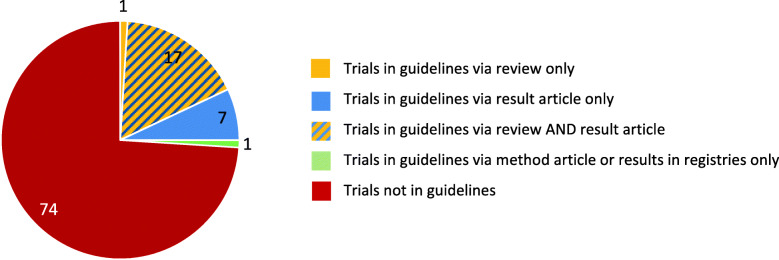
Proportion of trials cited by guidelines, shown by type of publication

We found 574 citations of 178 trials (26%) in guidelines (Fig. 6). Some of the guidelines included information from several of our trials. These corresponded to 427 unique guidelines. On average, each of our trials was cited 3.2 times (574/178) in guidelines. This “guideline inclusion factor” ranged between 2.9 and 3.7 for the sub-cohorts.

One trial can be included in one guideline via several pathways, namely via a published article or via a systematic review. The following analysis shows via what publication type trials were included in guidelines: 69% (122 of 178) of the trials were included in 285 of 427 (67%) different guidelines via the citation of 382 systematic reviews. 58% (104 of 178) of the trials were included directly in 226 of 427 (53%) different guidelines via the citation of 262 result articles. In total, 93% (166 of 178) of the inclusions in guidelines come from result articles via a direct or indirect pathway. 6% (10 of 178) of the trials were included in 12 of 427 (3%) different guidelines via citation of 12 method articles. 4% (7 of 178) of the trials were included in 6 (2%) different guidelines via citation of seven registry information (Table 5).

**Table 5:** Proportion of trials (*n* = 691) cited by clinical guidelines per sub-cohort and type of publication

Trials cited by guideline	IIT Public Germany gov No. of trials (%)	IIT Public Germany other No. of trials (%)	IIT Public Germany (total) No. of trials (%)	IIT Public International No. of trials (%)	IST Commercial Germany No. of trials (%)	IST Commercial International No. of trials (%)	Total No. of trials (%)
**Total trials**	60	60	120 (100)	200 (100)	171 (100)	200 (100)	691 (100)
Trials in guidelines	27 (45)	8 (13)	35 (29)	61 (31)	50 (29)	32 (16)	178 (26)
95% CI	32–58	6–25	21–38	24–37	23–37	11–22	23–29
**Direct**							
Trials with method articles in guideline	7 (12)	0	7 (6)	2 (1)	0	1 (< 1)	10 (1)
Trials with result articles in guideline	19 (32)	4 (7)	23 (19)	36 (18)	27 (16)	18 (9)	104 (15)
Trials with register ID in guidelines	3 (5)	0	3 (3)	2 (1)	2 (1)	0	7 (1)
Trials with any direct citation	25 (42)	4 (7)	29 (24)	38 (19)	29 (17)	18 (9)	114 (16)
**Indirect**							
Trials in guidelines via review	16 (27)	8 (13)	24 (20)	43 (22)	35 (20)	20 (10)	122 (18)
**Direct AND indirect**							
Trials in guidelines via review AND result article	12 (20)	8 (13)	20 (17)	42 (21)	35 (20)	20 (10)	117 (17)

Direct: guidelines cite the original published article(s); Indirect: guidelines cite systematic review(s) that include the original published article(s)

##### Sub-cohorts

**Fig. 7:**
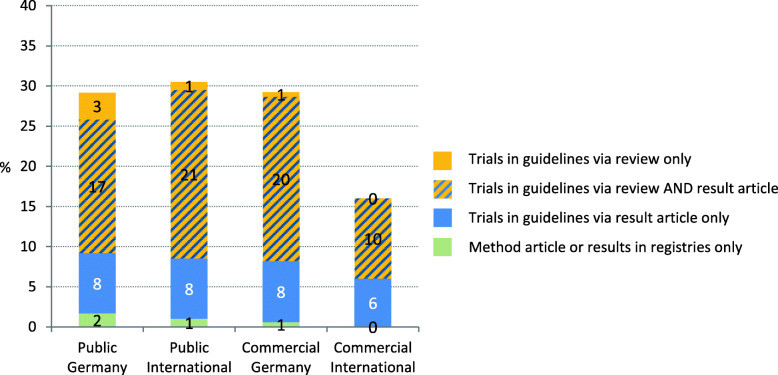
Proportion of trials with research impact per sub-cohort (*n* = 691). Trials included in a guideline via citation of a published article, of results published in registries or of a systematic review citing the trial

In Fig. 7 / Table 5 it is shown that for the sub-cohorts the inclusion of trials in guidelines ranged between 17 and 31%. For the subgroup Public Germany gov, even 45% (27 of 60) of the trials were cited in guidelines. Compared to Public Germany trials, the proportion of trials included in a guideline is similar to Public International and Commercial Germany trials, whereas commercial International trials are less often included in guidelines.

#### Study characteristics associated with inclusion in a guideline

Similar to the inclusion in reviews, the multivariable analysis confirmed a significantly lower representation of Commercial International trials in guidelines compared to the other sub-cohorts and demonstrated that type of intervention and number of primary outcomes are not associated with the inclusion in guidelines. Larger trials are about twice as often included in guidelines than smaller trials (Additional file 10).

#### Overall impact

##### Lifecycle of trials

**Fig. 8:**
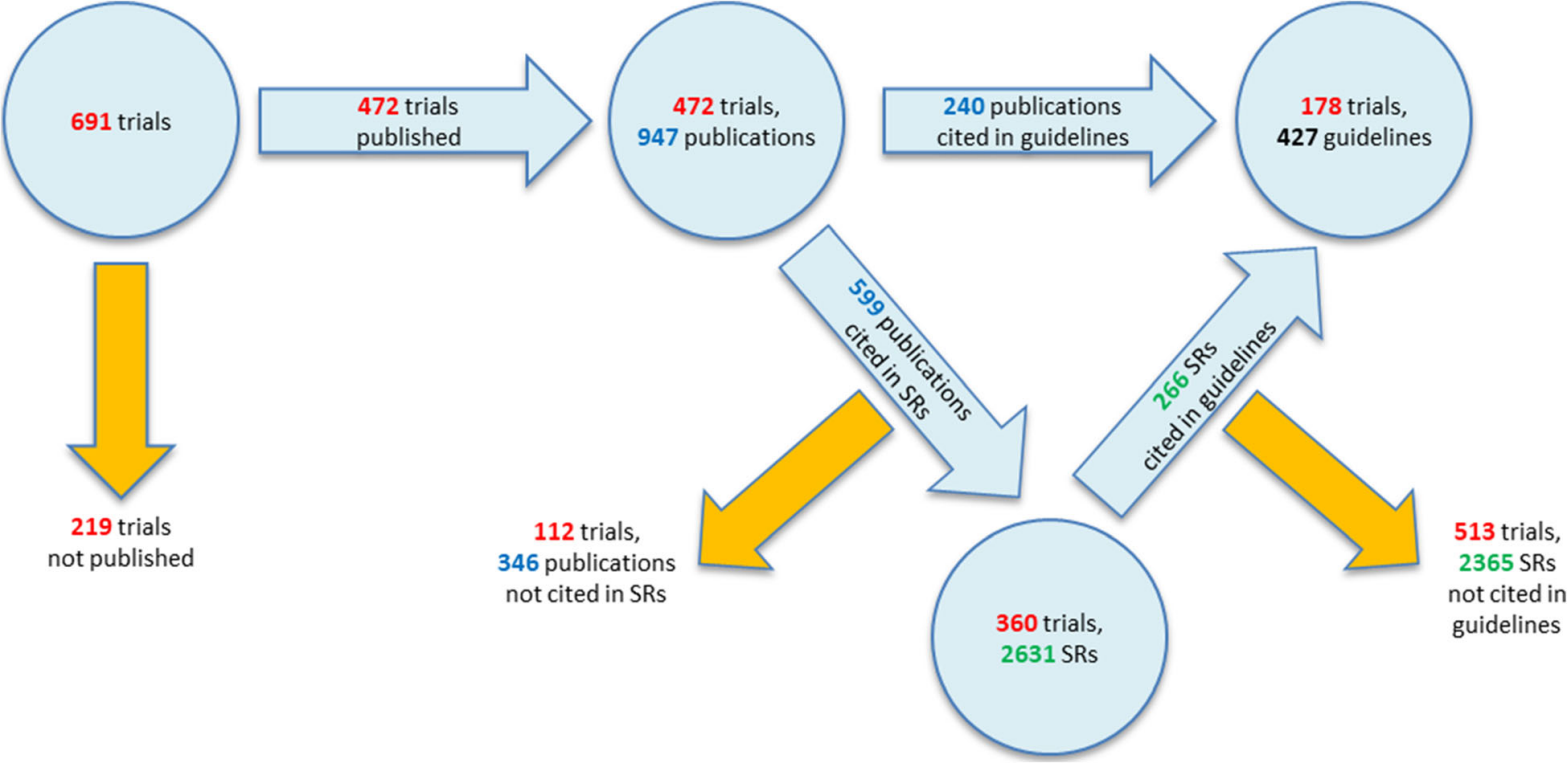
Impact on clinical practice. Total number of trials, published articles and systematic reviews (SRs) and guidelines, citing the published articles

Figure 8 shows the fate of the trials included in our cohort from registration to publication and to their impact on clinical practice. During their lifecycle from registration to impact in clinical practice, the number of relevant trials decreases with each step. 17% of the trials have no published results. Of the 576 (83%) published trials, 15% (107 of 691) have their results only published in registries and therefore might have less awareness and a limited impact in the scientific community.

Trials published as journal article(s) (472; 68%) have a good chance to be cited in reviews or guidelines. Nevertheless, in our cohort, a relevant percentage did not find an inclusion in clinical practice: only 309 (45%) of the trials were included in systematic reviews and 178 (26%) in guidelines.

##### Cohort

**Fig. 9:**
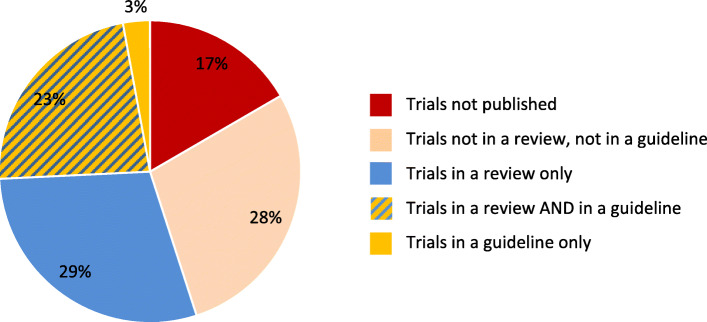
Publication and impact of trials

Of all trials, 274 (40%) generated no impact: 115 (17%) of the trials were not published and of the published trials, 160 (23%) were not cited by either a systematic review or a guideline (Fig. 9).

Used by secondary research articles were 417 (60%) trials: 361 (52%) were cited by a systematic review, and 178 (26%) by a guideline. Out of those, 123 (18%) were cited by both, a systematic review and a guideline. This means that more than half (52%) of the trials were cited in a systematic review and that about a quarter (26%) reached an impact in a clinical guideline.

##### Sub-cohort

**Fig. 10:**
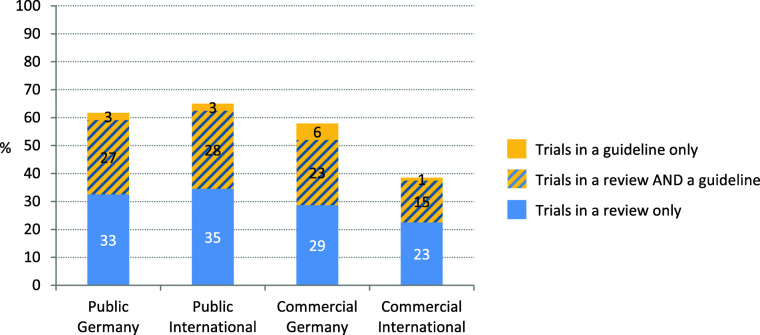
Impact of trials per sub-cohort

Commercially funded trials, especially Commercial International trials, less often gain an impact by inclusion in systematic reviews (52%; 39% for SRs, and 29%; 31% for guidelines) than publicly sponsored trials or guidelines (59%; 63% for SRs, and 29 and 17% for guidelines). The distribution of the three “impact-proportions” concerning inclusion in reviews and/or guidelines showed only minor differences between the sub-cohorts (Fig. 10).

#### Medical fields

**Fig. 11:**
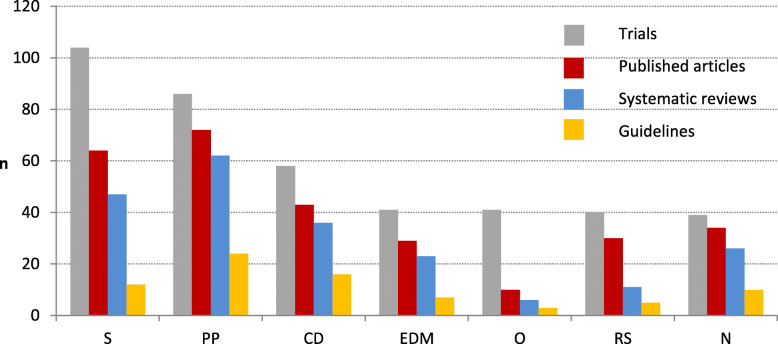
Fate of trials per medical fields: surgery (S), psychiatry and psychotherapy (PP), cardiovascular disease (CD), endocrinology, diabetes, and metabolism (EDM), Ophthalmology (O), respiratory system (RS), neurology (N)8

For our cohort, we found clear differences regarding the publication and impact for the main medical fields (number of trials ≥39). The high proportion of guidelines in psychiatry and psychotherapy, cardiovascular disease and neurology is related to a high proportion of publications and systematic reviews for these fields (Fig. 11). When publication is low, this results in fewer reviews and guidelines (ophthalmology, surgery).

## Discussion

In the current project we assessed the research impact on clinical practice of publicly sponsored trials and commercially sponsored trials conducted in Germany in comparison to those conducted internationally. By using a prospective strategy that followed the lifecycle of a defined set of trials from their registration up to their inclusion in systematic reviews and clinical guidelines, we have collected and analyzed data not only for those trials that were ‘successful’, but also for trials that were not published and did not gain an impact on clinical practice. We were also interested in trial characteristics that were associated with impact. Systematic reviews have shown that several study factors are associated with publication of clinical trial results, e.g. direction of study findings, study size and duration [4, 31]. Those factors could also have an influence on the impact measures that go beyond publication, and also study characteristics such as phase of clinical research (study phase), medical field, type of funding or sponsorship, and place of conduct (country of study sites) could play a role. An increased awareness of the risk factors could improve the feasibility and efficiency of future trials and the validity of trial results. Trials at high risk of having no impact could be adjusted beforehand to ensure a successful trial progress.

### Interpretation of findings

We compared IITs with ISTs because they often focus on different clinical questions and pursue different aims and objectives. IITs play a crucial role in academic clinical research whereas ISTs usually focus on commercial interests, mainly of pharmaceutical companies, whose primary aim is to develop and approve drugs or other medical treatments [32]. In IITs, an academic investigator is responsible for the conduct of the clinical trial, which includes planning, registration and publishing the results of the study [33]. IITs are often conducted to expand product knowledge, including safety, and to identify new ways of using existing treatments, which might lead to the improvement of patient health [34]. IITs complement ISTs regarding the medical field, such as physio- and psychotherapy, behavioral changes as well as complementary medicine.

Compared to previous research, in our project a high proportion of trials (83%) were published. Systematic reviews and retrospective research projects investigating the publication proportion of RCTs resulted in considerably lower proportions of 60 to 71% [4, 35–37]. Only 37% of RCTs presented as conference abstracts were published in full as journal articles [5]. The relatively high proportion of published trials and trial results in our cohort can be attributed to the fact that we also considered a trial as published, when its results were reported in a study registry. Results in registries were also considered in a recently published project that investigated the publication proportion of trials conducted at German university medical centers. Also in this project, a publication proportion of 83% for completed trials could be shown [38]. Subgroup analyses of this project also confirmed our results, that larger trials are more often published than smaller trials. A relatively high publication proportion of 73% was also found for completed academic drug trials approved by the Danish Medicines Agency [39]. However, academic trials approved by an Ethics committee in Spain had a considerable lower publication proportion of 39%, whereas 64% of the commercially sponsored trials were published in a peer-reviewed scientific journal [40]. For comparison, the publication proportion of research projects beyond clinical trials, e.g. basic research, funded by a medical faculty in Germany, was 65% for publication in a peer-reviewed journal, and 73% if also other publications were counted [41].

Even though there are several advantages for posting results in registries, e.g. results can be presented fast and concisely, they are directly attached to the registry record, and provide information about the trial methods as well as references and links to further trial information, the publication proportion is still relatively small. In a cross sectional study across academic medical centers in the United States, the publication proportion of completed trials that were registered in ClinicalTrials.gov was analyzed. Across the medical centers, 10.8 to 40.3% of the trials were published within 24 months of study completion, and for 1.6 to 40.7%, results were reported on ClinicalTrials.gov [42].

In our cohort, on average, for 45% (range of sub-cohorts: 36–68%) of the trials we found results in study registries. This finding is in line with the results of a recent study, investigating the compliance with the Food and Drug Administration Amendments Act of 2007 (FDAAA) concerning reporting of trial results. The researchers found, that due to report results under the FDAAA only about 40% of all applicable trials reported their results in ClinicalTrials.gov within the 1 year deadline after study completion [43].

The possibility to add results to a study record in registries, is certainly an important step to improve transparency in clinical research. However, limited, incomplete or expired trial information in registries often make it difficult to get a complete picture of the trial and to appraise and interpret the results. Several initiatives such as AllTrials and TranspariMed work on the improvement of a trial’s reporting by requiring clinical trials to be registered and to report their full methods and summary results [14, 22].

In our cohort, of the trials with published results, 19% (107 of 556) were solely available in study registries. This has serious implications for the search process to identify relevant studies, i.e. which sources need to be searched, especially for systematic reviews and clinical guidelines. A search strategy should not only focus on journal articles, but should be accomplished by an additional search in study registries- This has already become mandatory for conducting Cochrane intervention reviews [44, 45]. To improve the findability of trial results, the registries themselves should improve their searchability. They should be constructed in a standardized format so that they are easily and reliably searchable, e.g. similar to biomedical databases by title, author, keywords and abstracts. Looking at our sub-cohorts, we found a significant difference between IITs and ISTs. For publicly sponsored trials, only 2–4% were solely published in registries, whereas this was the case for 20 to 32% of the commercially sponsored trials. A similar relation was found in a project investigating more than 30,000 clinical trials registered in the EU Clinical Trials Register (EUCTR) [46]. Of those trials that were due, which means that 12 months to publish the results had passed, about 50% reported results in this register; 68% of the commercial trials and only 11% of the non-commercial trials. This higher proportion of results in registries compared to our results could be explained by the fact that in EUCTR only trials investigating medicinal product are included and that for those trials disclosing of their results has been required by the European Medicines Agency (EMA) since 2004. A higher proportion of trials with results in registries were also shown for clinical trials sponsored by the pharmaceutical industry trials compared to non-commercially sponsored trials in Spain [40]. It must be noted that in this project the commercial trials were registered significantly more often in ClinicalTrials.gov than the academic trials.

The reasons for these observed differences are unclear and future analyses would be worth to compare the characteristics and results of those trials published solely in registries with those published as journal articles, e.g. regarding publication bias. One explanation could be that for publicly funded trials publication of results in the form of a journal article is often demanded by the funding organization and is part of the funding conditions. Advantages of publishing results as a journal article ideally are a quality-assured peer-review, trial methods and results are considered and discussed in the context of the existing evidence and can be commented by other researches e. g. via response letters. For a great proportion of trials in our cohort, results were published as journal articles (81%), and for more than half of the trials results were included in a registry.

Disclosure of detailed trial methods of a trial is essential with respect to the critical appraisal and interpretation of the results, and is the basis to enable other researchers to reproduce the trial and verify its results, which is a basic requirement for later implementation in medical practice. While in an original journal research article both methods and results of a trial are described, it is becoming more common to publish articles only describing the detailed methods of a trial and not the results. In our cohort, this was the case for 14% of the trials, for 3% only a method article could be identified. Moreover, it is important to point out that most of the method articles derive from publicly funded trials (87%), of which most of the German IITs were from Public Germany gov (67%). In scientific research it is not unusual to publish results of one study in more than one article. One reason for this could be that in academia the reward system is often built on quantity of research output [47]. Scientific success, such as reputation, career advancement, as well as successful applications for research funding, is directly associated with the publication output of a researcher. In our project, multiple publication was the case for 188 (40%) trials. They were more common for IITs (Germany: 33%, International: 32%) than for ISTs (Germany: 17%, International 14%). The trial with the highest number of identified publications (*n* = 21) was a phase 4 study, conducted in the field of cardiovascular disease, funded by the DFG. For this trial, one method article, two result articles and 18 sub-studies and secondary analysis were published. This trial and also the other high-frequently published trials (25 with more than 5 published articles) were conducted in academia. For this publication frequency, measured as the number of published articles per trial, we found a remarkable phenomenon: about one third of all published articles corresponded to only 8 % of the trials. Even though this aforementioned reward system and its consequences have been in the focus of criticism for several years, structures have still not changed [48].

In contrast, in industry the main (financial) interest lies in the results, i.e. efficacy and safety of the tested treatment, whereas the study protocol and methods used are often confidential to protect commercial interests. This is also shown by the public availability of the study protocols: for 40 trials of our cohort, the original study protocol could be identified, 30 belonged to the IIT sub-cohorts and 10 to the IST sub-cohorts.

A Health Technology Report was conducted to evaluate the impact of Cochrane Reviews published by 20 Cochrane Review Groups, on health care, patient outcomes and value for money [49]. Therefore, a random sample of 20 Cochrane Reviews and 40 selected reviews, more likely to have had an impact, were selected. Of the 60 included reviews, a considerable proportion of 67% had been cited in clinical guidance and 15 had influenced further primary research.

We found that more than half of the trials are represented in systematic reviews and more than a quarter in guidelines. To further improve this knowledge transfer from research into practice, several issues have to be considered. The first issue is to understand, why 17% of the trials have not been published. The second issue is how to improve the transfer of the 28% of the trials that were published but reached no impact.

Typical reasons for not publishing trial results as presented in a systematic review are lack of time and/or resources, non-completion of study, publication was not an aim, or only had low priority [50]. Further reasons stated by sponsors of Danish academic clinical drug trials were negative or not statistically significant results [39]. Possible explanations for published trial results not being included in systematic reviews are that no review related to the research question has been conducted or updated after the date of publication. Reasons reported for non-inclusion of published articles in the systematic reviews of our study cohort were that the eligibility criteria were not fulfilled, e.g. wrong patient group, intervention, comparator, outcome measure, or study type.

For inclusion of trial results in guidelines, the same reasons as for systematic reviews could apply. However, in guidelines, in addition to publications, systematic reviews are also a relevant pathway for inclusion of trial results. A detailed investigation of the systematic reviews that have not been included in guidelines (56%) would be useful, e.g. to find the reasons for lack of guidelines and to be able to further improve the transfer of important trial findings into medical practice.

### Strengths and limitations of the study

A strength of our study was that all trials were registered in study registries so that for all of them basic study information was available. Study characteristics were double-extracted independently in a pre-piloted extraction form following a written manual. All data extractors were trained prior to the data extraction. We captured all relevant information available in any study registry. Discrepancies in different sources were discussed and resolved. The identification of systematic reviews citing the original study report was conducted semi-automatically by using a self-developed program. The search for clinical guidelines was done manually following predefined standardized rules.

Another strength was that we controlled for possibly biasing factors by design, i.e. by balancing important study characteristics to Public Germany as the reference sub-cohort.

A limitation arising from this was the limited number of studies in the sub-cohorts Public Germany and Commercial Germany. The number of trials meeting our inclusion criteria for the Public Germany gov (reference sub-cohort) was fixed to 60. For the sub-cohort Commercial Germany, a balancing for non-drug trials was not fully possible: only 171 could be identified in DRKS and ClinicalTrials.gov registries instead of the pre-planned 200 studies per comparison sub-cohort. However, it is not expected that this difference of 29 trials have a relevant influence on our results.

Our cohort was composed of trials that were included in study registries and, partially, also in databases maintained by funding organizations. Against the background that still not all trials are registered, our trial cohort might be a “positive” selection compared to those conducted worldwide. Therefore, there is a potential risk that our cohort is biased, resulting in a limited external validity of our project results.

Even though all studies were included in at least one study registry, for some studies information in registries was scarce and detailed study protocols were only rarely available. Therefore, for some trials it was difficult to find out whether a published article corresponded to the trial. We also had to rely on the information reported in registries. Data of prospectively registered studies can include preliminary study information, for example information about study start and completion date. This may have influenced our findings.

We tried to assess actual data and included trials that started in 2005 or later and were completed by the end of 2016, for which we searched for corresponding publications in 2018/2019. For trials completed late during this time period, there might not have been sufficient time for publication and inclusion in systematic reviews and guidelines. Our results, however, indicate that this only concerns a few trials because since 1) compared to literature, the publication rate of our cohort was relatively high, and 2) the stacked probability plot (Fig. 3) also indicates that only few first publications are to be expected. Nevertheless, in such projects there will always be a compromise between presenting actual data with respect to the timeframe of included studies and leaving enough time for studies to be published and have an impact.

An important result of our study was that for 15% of the trials, results were solely available in study registries and were not published as journal article. In such cases, we could only search for guidelines citing the trial by using the registry identification number, but this was not possible for systematic reviews. To identify citing systematic reviews, we used the “cited by”- or “times cited”-functions of PubMed and WoS. These functions only consider journal articles, so that we were limited to the published journal articles.

The full text of some clinical practice guidelines from the United Kingdom identified via NICE or TRIP were only accessible to people located within the country, so that we were not able to verify the citation for those. Therefore, we did not consider them for our project.

## Conclusion

To the best of our knowledge, our project provides the first comprehensive and comparative evaluation of investigator-initiated trials and industry-sponsored trials with regard to their impact on clinical practice. It comprises not only publication of trial results in journals as well as in study registries but also examines the different factors that can be used as selective inclusion criteria of results in systematic reviews, meta-analyses and clinical guidelines. Previous investigations have focused on only one aspect of clinical impact, mostly on the publication of results. They also have been concentrated on a specific medical field or were based on studies conducted at a single institution. An encouraging result of this project is that with 83% a high proportion of clinical trials were published, which is a significant improvement compared to previous investigations. A reasonable percentage of trials were used in systematic reviews (52%) as well as in clinical guidelines (26%). IITs performed comparably or not significantly worse than ISTs with respect to the three IMPACT-aspects investigated. For publishing study results, study registries have become an important alternative or complement to journal articles. Nevertheless, there still were a certain proportion of trials that were not published and/or had no impact which needs to be decreased to increase the efficiency and to reduce waste in medical research. As a first step, special attention could be given to smaller trials and non-drug trials, which were significantly less often published or cited by systematic reviews or clinical guidelines than larger trials and non-drug trials. For statistical reasons, larger trials had a higher probability to generate statistically significant results. Nevertheless, trials with a smaller number of participants, e.g. as is the case in early phase studies or in case of limited scientific resources, are also justified and should be available for inclusion in the total body of evidence. Further research is needed to identify the reasons and risk factors for non-publication or delayed publication of registered trials and for non-citation in reviews and guidelines. A standardized reporting system, implemented in the lifecycle of studies, that requests the reasons in these cases could be an approach to reach that goal. Such a system could also reveal, why published results of commercial trials appear less often in reviews and guidelines than academic trials.

### Implications for policy, practice and research

Further efforts are needed to ensure that the results of all trials conducted are published. Publication of all results of all trials should become mandatory, e.g. by legal regulation and by requirement of funding organizations. The proportion of published results of commercial trials is comparable to those of academic trials, but they appear less often in reviews and guidelines. Further research is needed to investigate the reasons for this phenomenon. In this project, the only criterion for measuring impact of trials on medical practice was inclusion or exclusion of their results. A more detailed quantitative analysis of the “value” of their contribution to the overall body of evidence and on medical practice would be helpful to identify “valuable” trials. Considering their study characteristics in the planning of future trials, could increase the impact of clinical research. Specifically for Germany, IITs funded by governmental bodies reached an impact comparable to international IITs and ISTs that is respectable. Thus, funding of high-quality IITs by governmental bodies is worth the effort, should be continued and further encouraged.

## Supplementary Information

**Additional file 1:.** PRISMA flowcharts.

**Additional file 2:.** Sources where published articles were identified.

**Additional file 3:.** Number of published articles.

**Additional file 4: **Publication frequency: Proportion of trials (total: *n*=472) with n published method and results articles (total: *n*=947).

**Additional file 5:.** Study characteristics associated with publication of trial results.

**Additional file 6:.** Medical Fields.

**Additional file 7:.** Number of published articles cited by systematic reviews and/or by clinical guidelines per sub-cohort and type of publication.

**Additional file 8: **Citation frequency for published articles (*n*=599) by systematic reviews (*n*=2631).

**Additional file 9:.** Study characteristics associated with citation by systematic reviews.

**Additional file 10:.** Study characteristics associated with citation by guidelines.

## Data Availability

The datasets used and/or analyzed during the current study are available from the corresponding author on reasonable request. We will publish the data in the institutional repository of the University of Freiburg “FreiDok Plus” (https://freidok.uni-freiburg.de) in due course. The DoiScout – an automatic tool for gathering information about registered clinical trials and resulting publications is available on GitHub: https://github.com/kainitschke/doiscout.

## Abbreviations

AWMF: Arbeitsgemeinschaft der Wissenschaftlichen Medizinischen Fachgesellschaften (Association of the Scientific Medical Societies). https://www.awmf.org/leitlinien/leitlinien-suche.html
BMBF: Bundesministerium für Bildung und Forschung (Federal Ministry of Education and Research). https://www.bmbf.de
Cochrane Library: https://www.cochranelibrary.com/
DFG: Deutsche Forschungsgemeinschaft (German Research Foundation). https://www.dfg.de/
DOI: Digital Object Identifier
DRKS: Deutsches Register Klinischer Studien (German Clinical Trials Register). https://www.drks.de
Epistemonikos: Collaborative, multilingual database of health evidence and largest source of systematic reviews relevant for health-decision making, and of other types of scientific evidence. https://www.epistemonikos.org/
EUCTR: EU Clinical Trials Register. https://www.clinicaltrialsregister.eu/
EudraCT: European Union Drug Regulating Authorities Clinical Trials Database. https://www.clinicaltrialsregister.eu/ctr-search/search
EMA: European Medicines Agency. https://www.ema.europa.eu
GEPRIS: German Project Information System. Online database made available by the DFG that provides information on current DFG-funded research projects. https://gepris.dfg.de/gepris/OCTOPUS
Google scholar: Web search engine providing scholarly literature. https://scholar.google.com
IITs: Investigator Initiated Trials
ISRCTN registry: International Standard Randomized Controlled Trials Number registry. http://www.isrctn.com
ISTs: Industry Sponsored Trials
LIVIVO: Interdisciplinary search engine for literature and information in the field of life sciences, run by ZB MED – Information Centre for Life Sciences. https://www.livivo.de
Medline: Medical Literature Analysis and Retrieval System Online. Bibliographic database of life sciences and biomedical information. Accessible e.g. via the search engine PubMed
NCT: National Clinical Trial (number)
PubMed: Free search engine accessing primarily the MEDLINE database. https://www.ncbi.nlm.nih.gov/pubmed/
RCT: Randomized controlled trial
SR(s): Systematic review(s)
TRIP: Turning Research Into Practice. https://www.tripdatabase.com/
Web of Science: https://apps.webofknowledge.com
