# Supplementary material for: Investigator initiated trials versus industry sponsored trials - translation of randomized controlled trials into clinical practice (IMPACT)
Source: BMC Med Res Methodol. 2021 Aug 31;21:182. doi: 10.1186/s12874-021-01359-x (PMC8406615; doi:10.1186/s12874-021-01359-x)

Additional file 1: PRISMA Flowcharts

IIT Public Germany gov

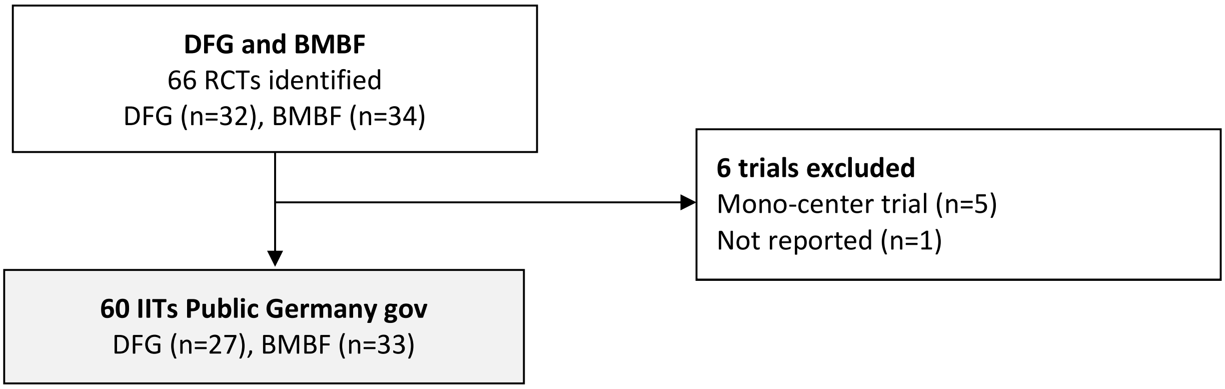

IIT Public Germany other

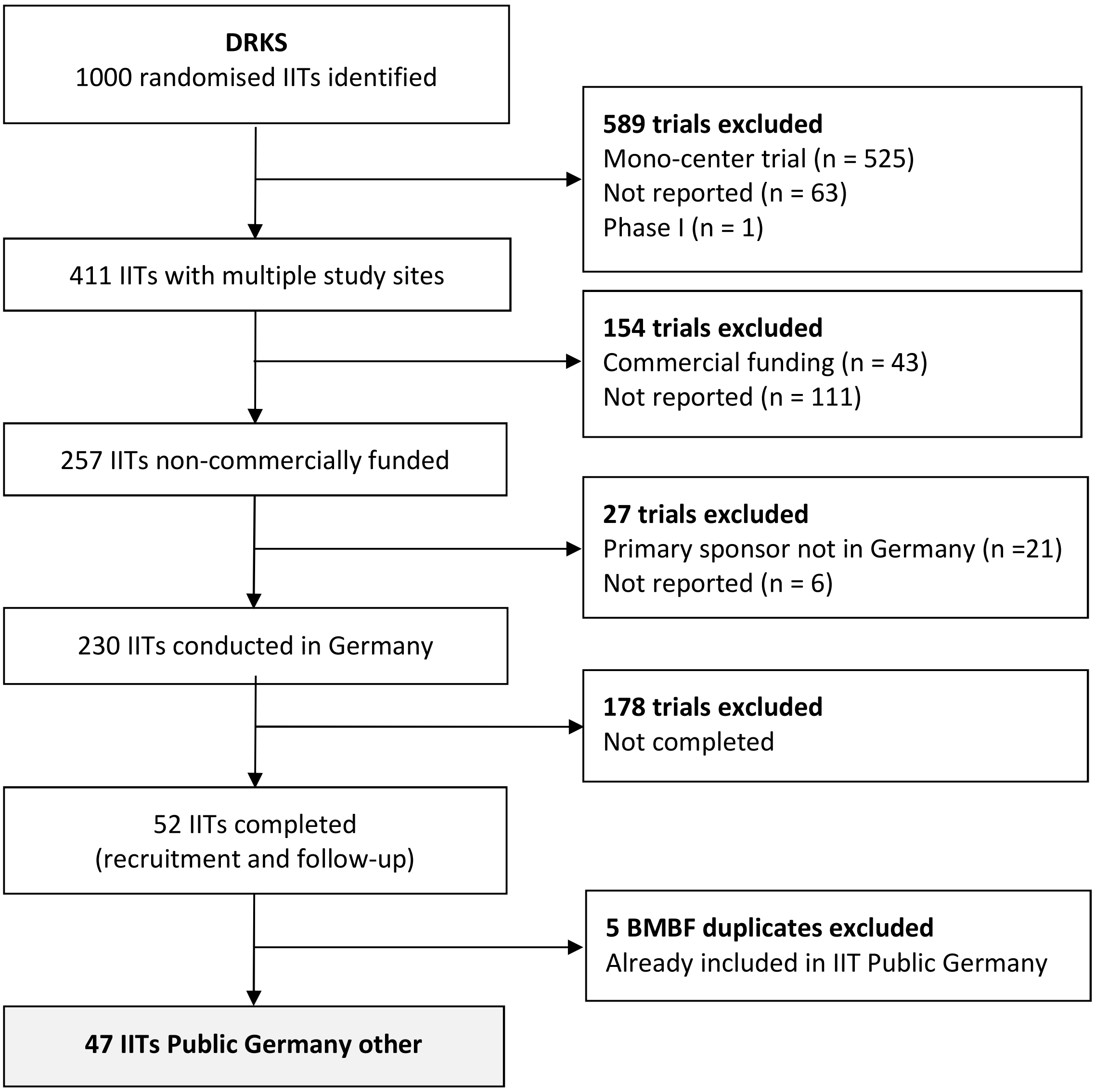

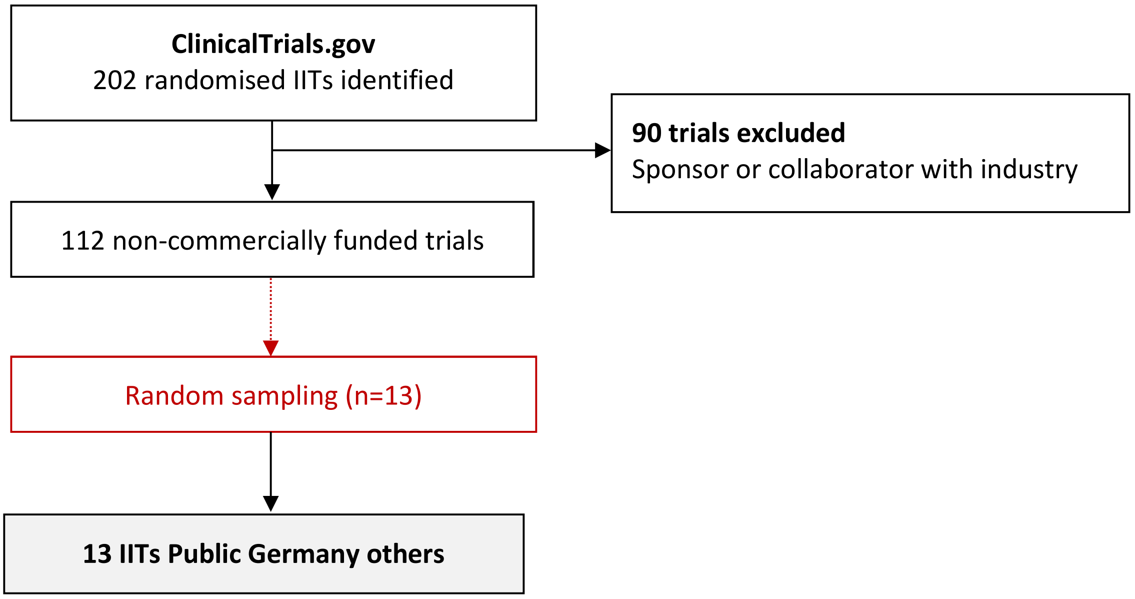
ClinicalTrials.gov
202 randomised IITs identified
90 trials excluded
Sponsor or collaborator with industry
112 non-commercially funded trials
Random sampling (n=13)
13 IITs Public Germany others

IIT Public International

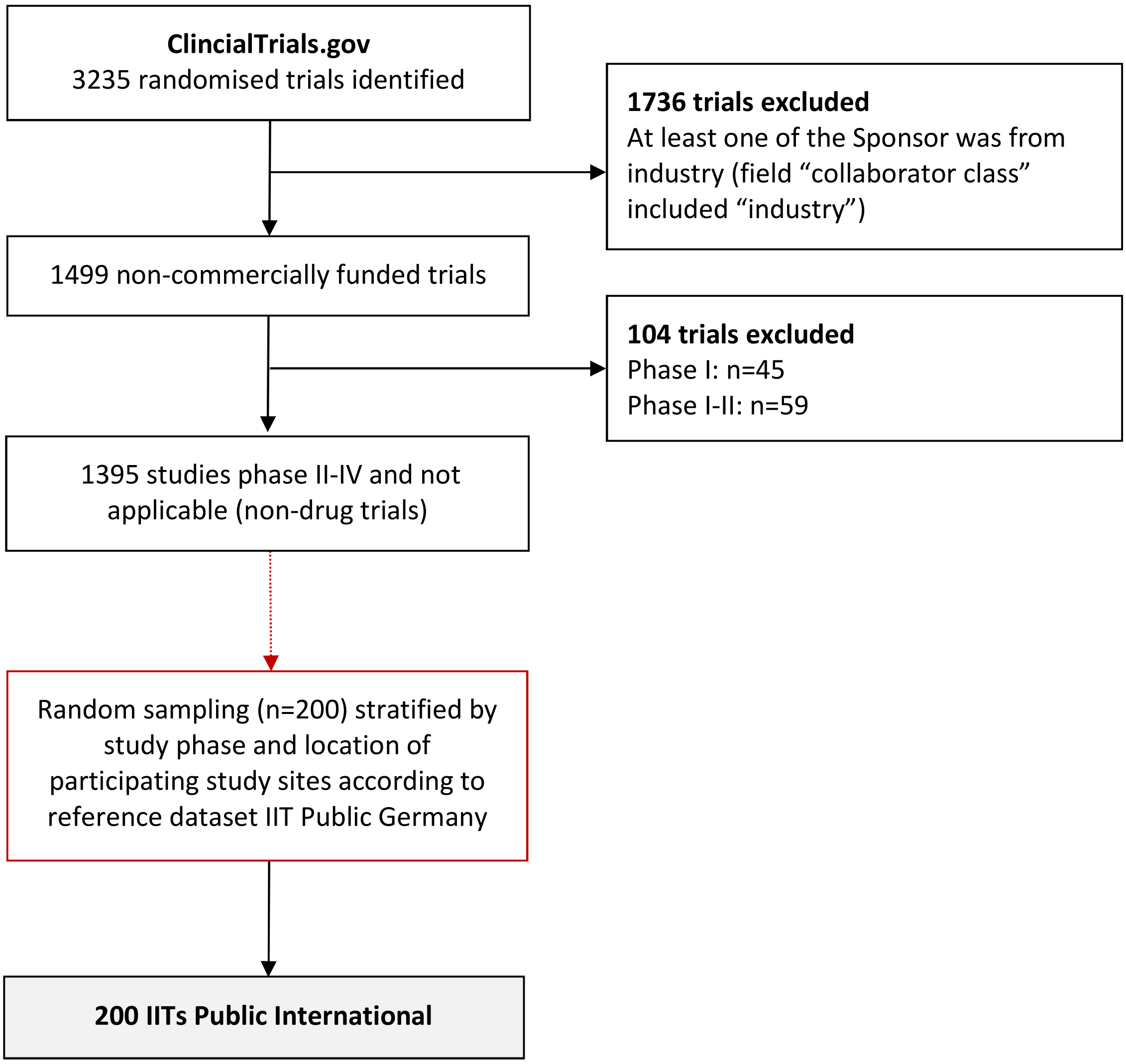

IST Commercial Germany

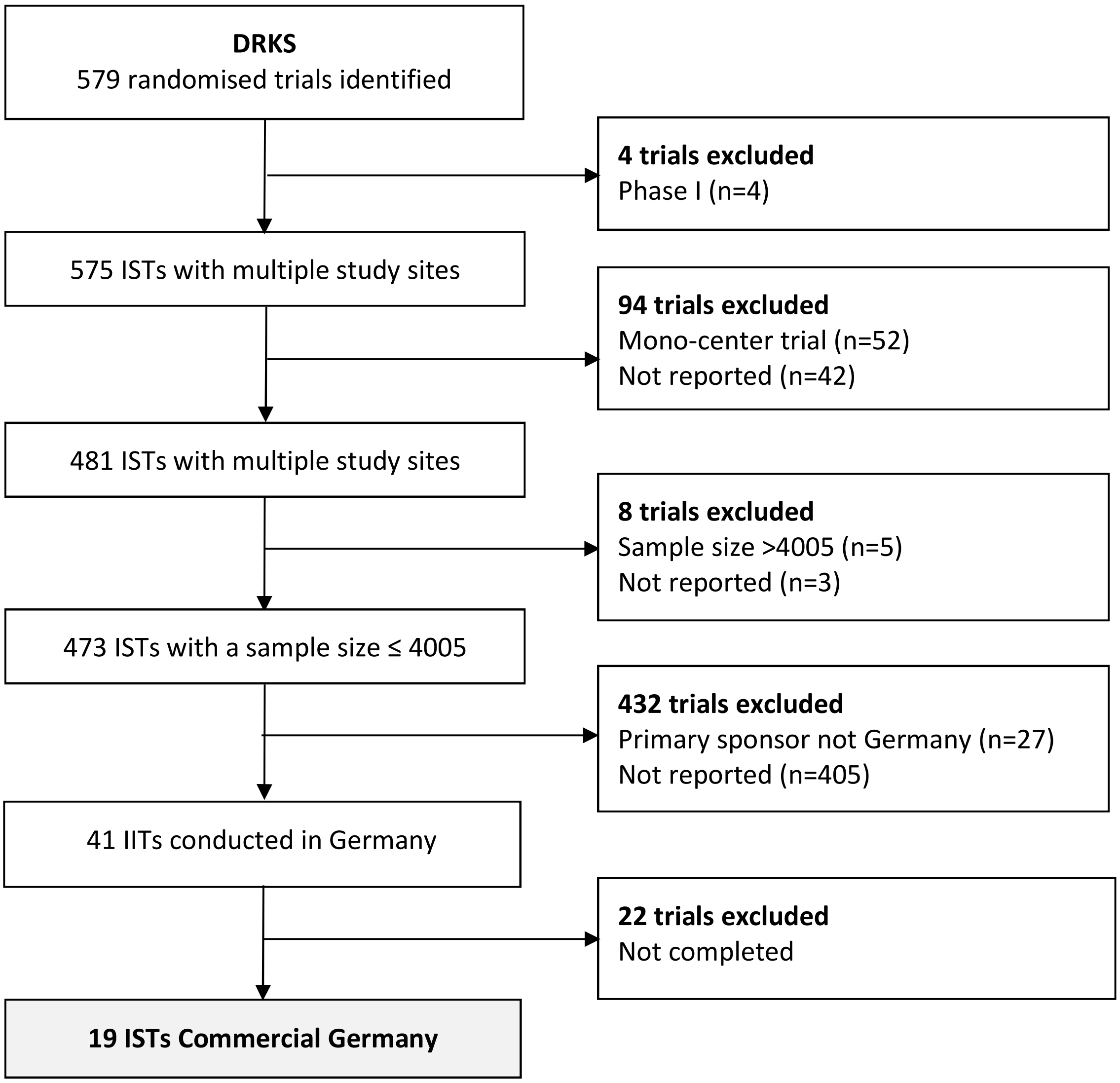

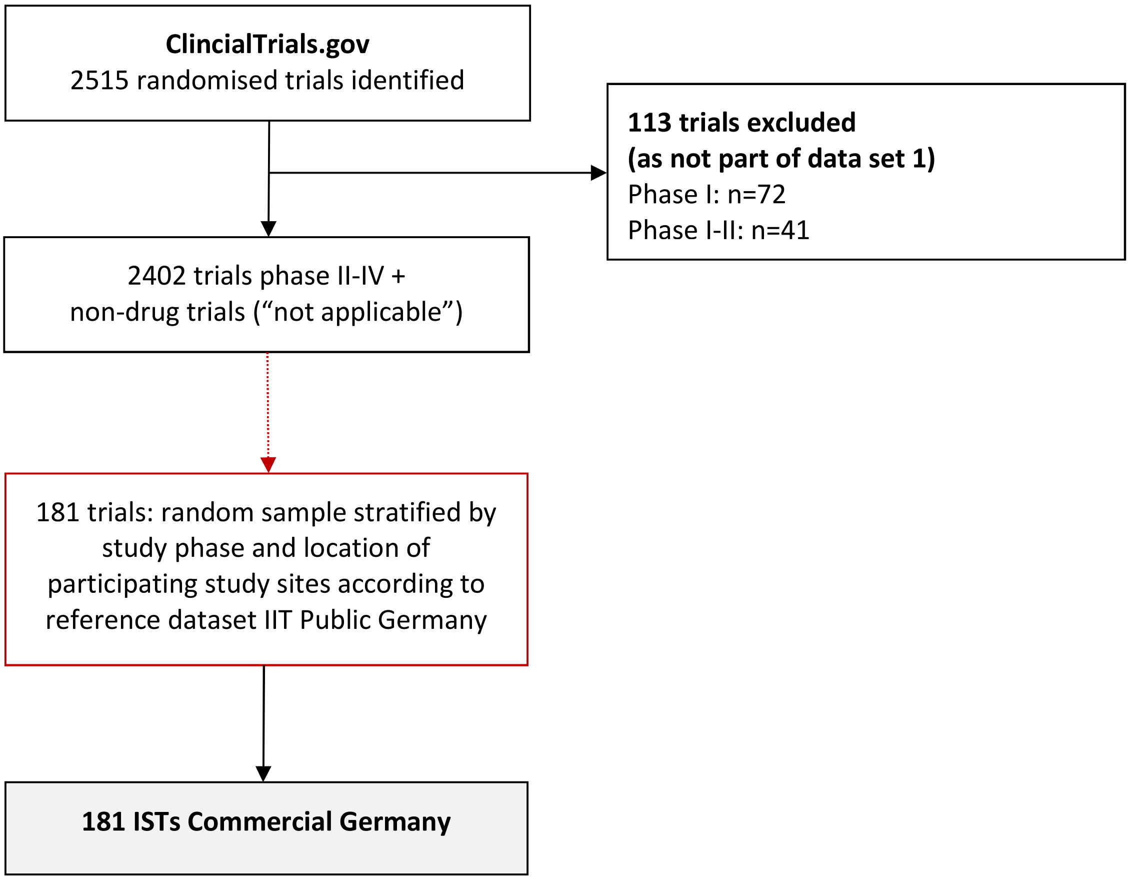
ClincialTrials.gov
2515 randomised trials identified
113 trials excluded
(as not part of data set 1)
Phase I: n=72
Phase I-II: n=41
2402 trials phase II-IV +
non-drug trials ("not applicable")
181 trials: random sample stratified by study phase and location of participating study sites according to reference dataset IIT Public Germany
181 ISTs Commercial Germany

IST Commercial International

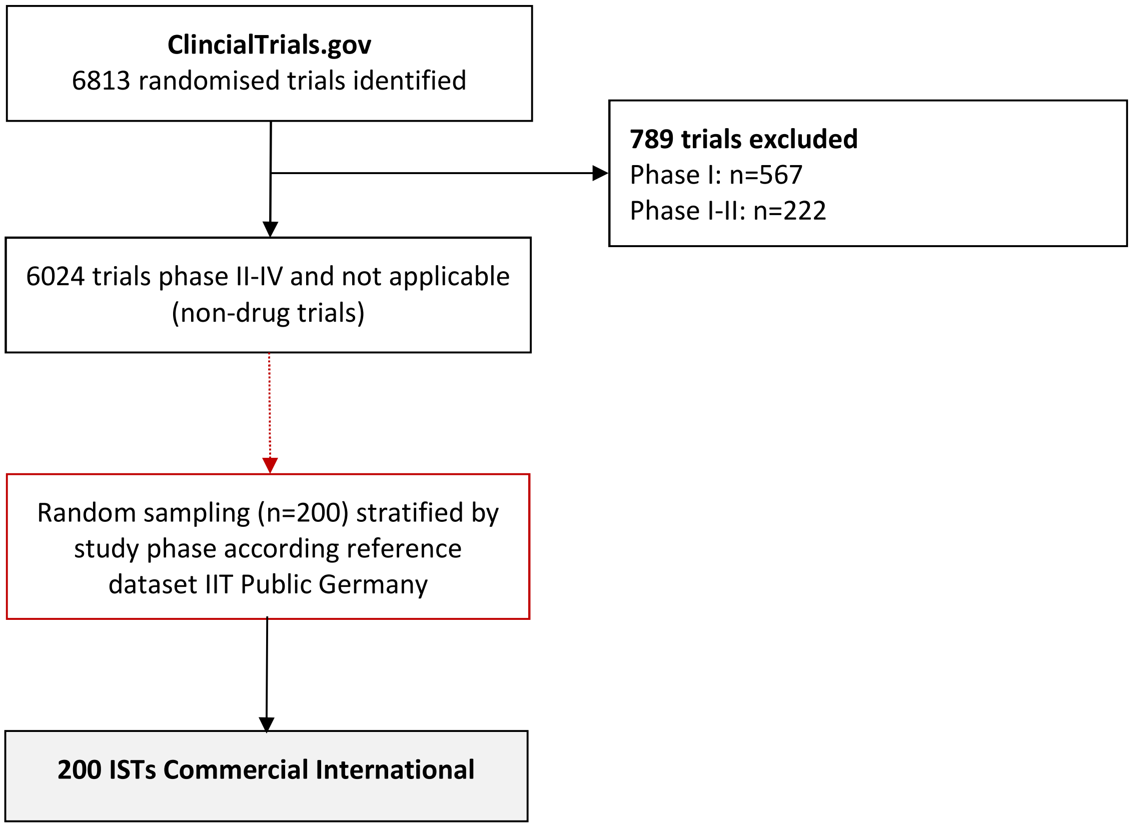

Supplement: Supplementary file 1 — Additional file 1:. PRISMA flowcharts. [file 12874_2021_1359_MOESM1_ESM.pdf]
