# Supplementary material for: Investigator initiated trials versus industry sponsored trials - translation of randomized controlled trials into clinical practice (IMPACT)
Source: BMC Med Res Methodol. 2021 Aug 31;21:182. doi: 10.1186/s12874-021-01359-x (PMC8406615; doi:10.1186/s12874-021-01359-x)
Supplement: Supplementary file 2 — Additional file 2:. Sources where published articles were identified. [file 12874_2021_1359_MOESM2_ESM.pdf]

Additional file 2: Sources where published articles were identified

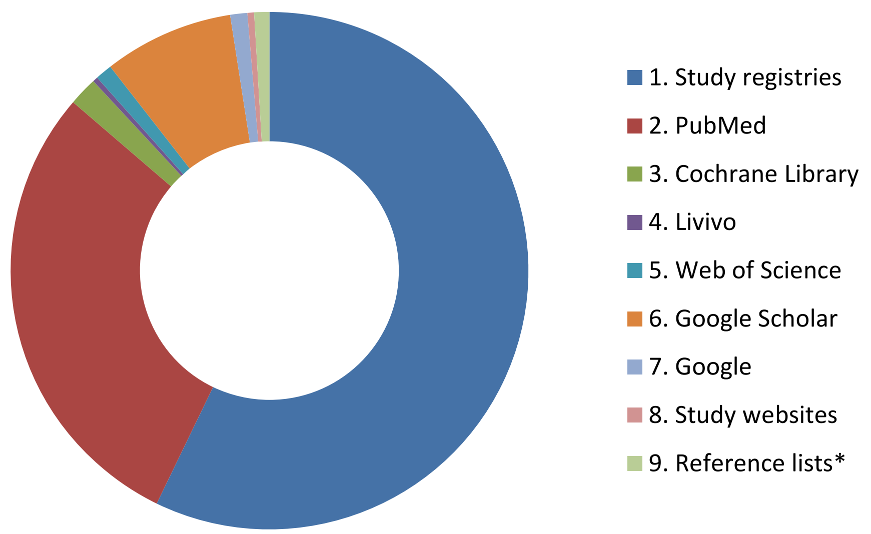


Number of publications identified in biomedical databases and other sources by using an incremental search strategy from 1. to 9. * including PubMed tools "Similar articles" and "Cited by".

Of the 947 identified published journal articles, a great proportion (894; 94%) was identified in three sources only: the references of more than half (541; 57%) of the identified articles were reported in the study registries, additional 276 (29%) were found via PubMed, and a Google Scholar search revealed another 77 (8%) articles. The incremental benefit of the other databases was marginal. The remaining 53 articles were identified in the Cochrane Library (17), Livivo (3), via Google (10), Web of Science (10), study websites (4) and PubMed tools "Similar articles" and "Cited by" and reference lists (9).

It must be emphasized that the proportion of identified articles in each source strongly depends on the sequence the sources were searched, as the article was assigned to the source were it was found first.
