# Supplementary material for: Investigator initiated trials versus industry sponsored trials - translation of randomized controlled trials into clinical practice (IMPACT)
Source: BMC Med Res Methodol. 2021 Aug 31;21:182. doi: 10.1186/s12874-021-01359-x (PMC8406615; doi:10.1186/s12874-021-01359-x)
Supplement: Supplementary file 3 — Additional file 3:. Number of published articles. [file 12874_2021_1359_MOESM3_ESM.pdf]

Additional file 3: Number of published articles

**Sub-cohort**	IIT Public Germany gov No. of articles (%)	IIT Public Germany other No. of articles (%)	**IIT Public Germany (total) No. of articles (%)**	**IIT Public International No. of articles (%)**	**IST Commercial Germany No. of articles (%)**	**IST Commercial International No. of articles (%)**	**Total No. of articles (%)**
**Total number of trials**	60	60	120	200	171	200	691
**Published articles total**	151 (16)	96 (10)	247 (26)	371 (39)	180 (19)	149 (16)	947 (100)
**Methods articles**	32 (21)	16 (17)	48 (19)	43 (12)	10 (6)	3 (2)	104 (11)
**Result articles**	119 (79)	80 (83)	199 (81)	328 (88)	170 (94)	146 (98)	843 (89)
