# Supplementary material for: Investigator initiated trials versus industry sponsored trials - translation of randomized controlled trials into clinical practice (IMPACT)
Source: BMC Med Res Methodol. 2021 Aug 31;21:182. doi: 10.1186/s12874-021-01359-x (PMC8406615; doi:10.1186/s12874-021-01359-x)

Additional file 4: Publication frequency: Proportion of trials (total: n=472) with n published method and results articles (total: n=947)

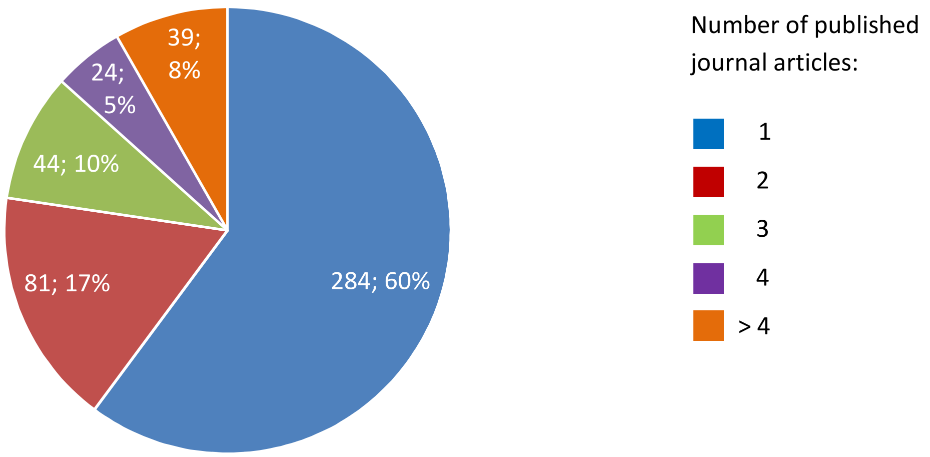

Supplement: Supplementary file 4 — Additional file 4: Publication frequency: Proportion of trials (total: n=472) with n published method and results articles (total: n=947). [file 12874_2021_1359_MOESM4_ESM.pdf]
