# Supplementary material for: Investigator initiated trials versus industry sponsored trials - translation of randomized controlled trials into clinical practice (IMPACT)
Source: BMC Med Res Methodol. 2021 Aug 31;21:182. doi: 10.1186/s12874-021-01359-x (PMC8406615; doi:10.1186/s12874-021-01359-x)
Supplement: Supplementary file 5 — Additional file 5:. Study characteristics associated with publication of trial results. [file 12874_2021_1359_MOESM5_ESM.pdf]

Additional file 5: Study characteristics associated with publication of trial results

**Covariates (number of trials)**	**Number (%) of published trials**	**Probability/ Odds ratio**	**95% CI**	**p-value**
Intercept (probability)		0.559	0.426-0.686	NA
IIT Public International (200)	170 (85)	4.300	2.455-7.654	0.0000
IST Commercial Germany (171)	147 (86)	4.316	2.400-7.941	0.0000
IST Commercial International (200)	163 (82)	2.896	1.703-4.968	0.0001
Non-drug trials (356) versus drug trials (335)	269 (75) versus 286 (85)	0.567	0.374-0.852	0.0068
Study size: n >150 (344) versus n ≤ 150 (346)	293 (85) versus 262 (75)	2.161	1.428-3.307	0.0003
Number of primary outcome(s): > 1 (165) versus 1 (525)	139 (84) versus 415 (79)	1.740	1.078-2.894	0.0275

Impact of the covariates on the probability of a study to be published. The second column contains in the first row the probability of being published for the intercept category and in the other rows the odds ratios for the other covariate categories.
