# Supplementary material for: Investigator initiated trials versus industry sponsored trials - translation of randomized controlled trials into clinical practice (IMPACT)
Source: BMC Med Res Methodol. 2021 Aug 31;21:182. doi: 10.1186/s12874-021-01359-x (PMC8406615; doi:10.1186/s12874-021-01359-x)
Supplement: Supplementary file 6 — Additional file 6:. Medical Fields. [file 12874_2021_1359_MOESM6_ESM.pdf]

Additional file 6: Medical Fields

Medical Fields	Total number of trials	Number of published trials (%)	Percent of published trials per medical field	95 % CI
Surgery	104	64	62	0.515-0.709
Psychiatry and Psychotherapy	86	72	84	0.742-0.908
Cardiovascular disease	58	43	74	0.610-0.848
Endocrinology, diabetes, and metabolism	41	29	71	0.545-0.839
Ophthalmology	41	10	24	0.124-0.403
Respiratory System	40	30	75	0.588-0.873
Neurology	39	34	87	0.726-0.957
Paediatric and Juenile Medicine	37	26	70	0.530-0.841
Hematology / oncology	34	25	74	0.556-0.871
Gynaecology and Obstetrics, Gynaecological Endocrinology and Reproductive Medicine	29	23	79	0.603-0.920
Dermatological and Venereal Diseases	28	17	61	0.406-0.785
Infectious disease	25	18	72	0.506-0.879
Physical and Rehabilitative Medicine	24	16	67	0.447-0.844
Nephrology/Urology	24	11	46	0.256-0.672
Gastro-intestinal	18	12	67	0.410-0.867
Family medicine	13	7	54	0.251-0.808
Orthopaedics	12	5	42	0.152-0.723
Rheumatology	11	7	64	0.308-0.891
Dentistry	10	7	70	0.348-0.933
Otorhinolaryngology	8	3	38	0.085-0.755
Allergy & Immunology	5	4	80	0.284-0.995
Pathology	2	2	100	0.158*-1.000
Anaesthesiology	2	1	50	0.013-0.987
Total	691	472	68	0.647-0.7176

*One-sided 97.5% confidence interval.

For each trial, we determined the medical field according to the slightly modified version of the medical fields specified in the “(Model) Specialty Training Regulations 2003” of the German Medical Association.
