# Supplementary material for: Investigator initiated trials versus industry sponsored trials - translation of randomized controlled trials into clinical practice (IMPACT)
Source: BMC Med Res Methodol. 2021 Aug 31;21:182. doi: 10.1186/s12874-021-01359-x (PMC8406615; doi:10.1186/s12874-021-01359-x)
Supplement: Supplementary file 7 — Additional file 7:. Number of published articles cited by systematic reviews and/or by clinical guidelines per sub-cohort and type of publication. [file 12874_2021_1359_MOESM7_ESM.pdf]

Additional file 7: Number of published articles cited by systematic reviews and/or by clinical guidelines per sub-cohort and type of publication

	IIT Public Germany gov No. of articles (%)	IIT Public Germany other No. of articles (%)	**IIT Public Germany (total) No. of articles (%)**	**IIT Public International No. of articles (%)**	**IST Commercial Germany No. of articles (%)**	**IST Commercial International No. of articles (%)**	**Total No. of articles (%)**
**Journal articles included in reviews**							
Method articles (total, n=104)	25 (24)	7 (7)	32 (31)	31 (30)	5 (5)	2 (2)	70 (67)
Result articles (n=843)	66 (8)	43 (5)	109 (13)	190 (23)	125 (15)	105 (12)	529 (63)
Journal articles total (n=947)	91 (10)	50 (5)	141 (15)	221 (23)	130 (14)	107 (11)	599 (63)
**Publications included in guidelines**							
**Journal articles**							
Method articles (n=104)	2 (2)	10 (10)	12 (12)	11 (11)	2 (2)	1 (1)	26 (25)
Result articles (n=843)	12 (1)	28 (3)	40 (5)	76 (9)	55 (7)	43 (5)	214 (25)
Journal articles total (n=947)	14 (1)	38 (4)	52 (5)	87 (9)	57 (6)	44 (5)	240 (25)
**Reviews**							
(n=2631)	42	23	65	110	69	67	286 (11)*

* The sum of the reviews in the sub-cohorts is higher than the total number of reviews, because one systematic review can cite published articles from more than one sub-cohort.
