# Supplementary figures and images for: Investigator initiated trials versus industry sponsored trials - translation of randomized controlled trials into clinical practice (IMPACT)
Source: BMC Med Res Methodol. 2021 Aug 31;21:182. doi: 10.1186/s12874-021-01359-x (PMC8406615; doi:10.1186/s12874-021-01359-x)

Additional file 8: Citation frequency for published articles (n=599) by systematic reviews (n=2631)

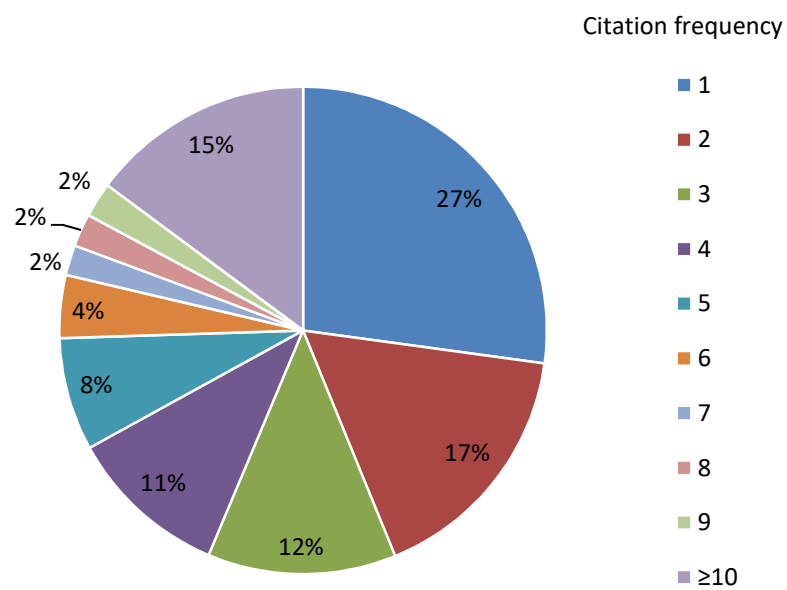

Supplement: Supplementary file 8 — Additional file 8: Citation frequency for published articles (n=599) by systematic reviews (n=2631). [file 12874_2021_1359_MOESM8_ESM.pdf]
