# Supplementary material for: Investigator initiated trials versus industry sponsored trials - translation of randomized controlled trials into clinical practice (IMPACT)
Source: BMC Med Res Methodol. 2021 Aug 31;21:182. doi: 10.1186/s12874-021-01359-x (PMC8406615; doi:10.1186/s12874-021-01359-x)
Supplement: Supplementary file 9 — Additional file 9:. Study characteristics associated with citation by systematic reviews. [file 12874_2021_1359_MOESM9_ESM.pdf]

Additional file 9: Study characteristics associated with citation by systematic reviews

**Covariates (number of trials)**	**Number (%) of published trials**	**Probability/ Odds ratio**	**95% CI**	**p-value**
Intercept (probability)		0.498	0.382-0.615	NA
IIT Public International (200)	125 (63)	1.357	0.838-2.196	0.2133
IST Commercial Germany (171)	89 (52)	0.824	0.505-1.341	0.4360
IST Commercial International (200)	75 (38)	0.407	0.252-0.652	0.0002
Non-drug trials (356) versus drug trials (335)	172 (48) versus 188 (56)	0.824	0.598-1.135	0.2362
Study size: n >150 (344) versus n ≤ 150 (346)	208 (60) versus 152 (44)	2.195	1.588-3.048	0.0000
Number of primary outcome(s): > 1 (165) versus 1 (525)	86 (52) versus 273 (52)	1.105	0.763-1.602	0.5979

Impact of the covariates on the probability of a study to be published. The second column contains in the first row the probability of being published for the intercept category and in the other rows the odds ratios for the other covariate categories.
