# Supplementary material for: Investigator initiated trials versus industry sponsored trials - translation of randomized controlled trials into clinical practice (IMPACT)
Source: BMC Med Res Methodol. 2021 Aug 31;21:182. doi: 10.1186/s12874-021-01359-x (PMC8406615; doi:10.1186/s12874-021-01359-x)
Supplement: Supplementary file 10 — Additional file 10:. Study characteristics associated with citation by guidelines. [file 12874_2021_1359_MOESM10_ESM.pdf]

Additional file 10: Study characteristics associated with citation by guidelines

**Covariates (number of trials)**	**Number (%) of published trials**	**Probability/ Odds ratio**	**95% CI**	**p-value**
Intercept (probability)		0.167	0.103-0.256	NA
IIT Public International (200)	61 (31)	1.384	0.825-2.347	0.2221
IST Commercial Germany (171)	50 (29)	1.226	0.717-2.110	0.4591
IST Commercial International (200)	32 (16)	0.463	0.263-0.810	0.0071
Non-drug trials (356) versus drug trials (335)	78 (22) versus 100 (30)	0.760	0.526-1.097	0.1427
Study size: n >150 (344) versus n ≤ 150 (346)	122 (35) versus 56 (16)	3.189	2.187-4.702	0.0000
Number of primary outcome(s): > 1 (165) versus 1 (525)	46 (28) versus 132 (25)	1.334	0.873-2.023	0.1775

Impact of the covariates on the probability of a study to be published. The second column contains in the first row the probability of being published for the intercept category and in the other rows the odds ratios for the other covariate categories.
